# A review on the role of cyclin dependent kinases in cancers

**DOI:** 10.1186/s12935-022-02747-z

**Published:** 2022-10-20

**Authors:** Soudeh Ghafouri-Fard, Tayyebeh Khoshbakht, Bashdar Mahmud Hussen, Peixin Dong, Nikolaus Gassler, Mohammad Taheri, Aria Baniahmad, Nader Akbari Dilmaghani

**Affiliations:** 1 Department of Medical Genetics, School of Medicine, Shahid Beheshti University of Medical Sciences, Tehran, Iran; 2 Men’s Health and Reproductive Health Research Center, Shahid Beheshti University of Medical Sciences, Tehran, Iran; 3 Department of Pharmacognosy, College of Pharmacy, Hawler Medical University, Erbil, Kurdistan Region Iraq; 4 Center of Research and Strategic Studies, Lebanese French University, Erbil, Kurdistan Region Iraq; 5 Department of Obstetrics and Gynecology, Hokkaido University School of Medicine, Hokkaido University, Sapporo, Japan; 6 Section of Pathology, Institute of Forensic Medicine, Jena University Hospital, Jena, Germany; 7 Urology and Nephrology Research Center, Shahid Beheshti University of Medical Sciences, Tehran, Iran; 8 Institute of Human Genetics, Jena University Hospital, Jena, Germany; 9 Skull Base Research Center, Loghman Hakim Hospital, Shahid Beheshti University of Medical Sciences, Tehran, Iran

**Keywords:** Cyclin dependent kinases, CDK, Cancer

## Abstract

The Cyclin-dependent kinase (CDK) class of serine/threonine kinases has crucial roles in the regulation of cell cycle transition and is mainly involved in the pathogenesis of cancers. The expression of CDKs is controlled by a complex regulatory network comprised of genetic and epigenetic mechanisms, which are dysregulated during the progression of cancer. The abnormal activation of CDKs results in uncontrolled cancer cell proliferation and the induction of cancer stem cell characteristics. The levels of CDKs can be utilized to predict the prognosis and treatment response of cancer patients, and further understanding of the function and underlying mechanisms of CDKs in human tumors would pave the way for future cancer therapies that effectively target CDKs. Defects in the regulation of cell cycle and mutations in the genes coding cell-cycle regulatory proteins lead to unrestrained proliferation of cells leading to formation of tumors. A number of treatment modalities have been designed to combat dysregulation of cell cycle through affecting expression or activity of CDKs. However, effective application of these methods in the clinical settings requires recognition of the role of CDKs in the progression of each type of cancer, their partners, their interactions with signaling pathways and the effects of suppression of these kinases on malignant features. Thus, we designed this literature search to summarize these findings at cellular level, as well as in vivo and clinical levels.

## Introduction

Cyclin-dependent kinases (CDKs) are a group of serine/threonine kinases with crucial roles in the regulation of cell cycle progression. The activity of these kinases is induced by cyclins. In fact, CDK/cyclin complexes control progression of the cell cycle in an orderly manner [1]. Emerging evidence suggest that CDKs and cyclins actively participate in the regulation of transcription, epigenetic mechanisms, metabolic processes and self-renewal capacity of stem cells [1]. Most notably, some of these functions are exerted in an independent manner from establishment of CDKs/cyclins complexes [1]. Another group of proteins, namely cyclin-dependent kinase inhibitors (CKIs) has been revealed to negatively regulate cyclin/CDKs. The main function of CDKIs is to obstruct cell cycle transition and suppress cell proliferation through inhibition of the enzymatic activity of CDKs. Inhibitor of CDK4 proteins and CDK-interacting protein/kinase inhibitory proteins belong to this group [2].

Defects in the regulation of cell cycle and mutations in the genes coding cell-cycle regulatory proteins result in unrestrained proliferation of cells leading to formation of tumors [3, 4]. Accordingly, modulation of activity of these proteins by therapeutic agents has been suggested as a promising strategy for treatment of cancers [5]. Successful introduction of these modalities into clinical settings needs proper recognition of the role of CDKs in the progression of each type of cancer, their interacting molecules and signaling pathways and the effects of suppression of these kinases on malignant features. Thus, we designed this literature search to summarize these findings at cellular level, as well as in vivo and clinical levels.

## Cyclin-dependent kinase 1 (CDK1)

### Cell line studies

A recent study has demonstrated that vitro that centromere protein F (CENPF) through interaction with CDK1 can increase G2/M-phase transition, enhance cell proliferation and possibly activate the anti-tumor effects of p53 in a human adrenocortical carcinoma cell line. Moreover, assessment of GSEA has verified involvement of CENPF in the G2/M-phase cell cycle and p53 signaling [6].

Expression of CDK1 has also been found to be increased in bladder cancer cells, parallel with over-expression of the long non-coding RNA (lncRNA) PVT1. Notably, suppression of PVT1 has decreased activity, proliferative potential, colony formation, migratory capacity, and invasiveness of bladder cancer cells. miR-31 binding sites have been reported in both PVT1 and CDK1 transcripts. Taken together, PVT1-mediated reduction of miR-31 could increase expression of CDK1 in bladder cancer cells to enhance their proliferative potential, migration, and invasion [7]. Another study has shown the role of CDK1 in phosphorylation of TFCP2L1 at Thr177 in embryonic stem cells of mice as well as human bladder cancer cells. Notably, this type of phosphorylation has a crucial role in pluripotency and cell cycle progression of stem cells through modulation of expression of developmental genes. CDK1/TFCP2L1 axis is also involved in the induction of stemness characteristics and tumorigenic ability of bladder cancer cells [8]. Treatment of bladder cancer cells with the protein kinase D (PKD) inhibitor CRT0066101 has suppressed proliferation of these cells. CRT0066101 treatment or PKD2 silencing has induced cell cycle arrest at the G2/M phase, diminished expressions of cyclin B1, CDK1 and levels of CDK1 phosphorylated at Thr161, while increasing p27Kip1 and CDK1 phosphorylated at Thr14/Tyr15. This protein kinase inhibitor has also decreased expression of Cdc25C, which dephosphorylates and induces activity of CDK1, while enhancing function of Chk1, which suppresses CDK1 activity through phosphorylation and inactivation of Cdc25C. Moreover, CRT0066101 could elevate expression of a number of proteins that inhibit activity of the CDK1/cyclin B1 complex [9].

**Fig. 1:**
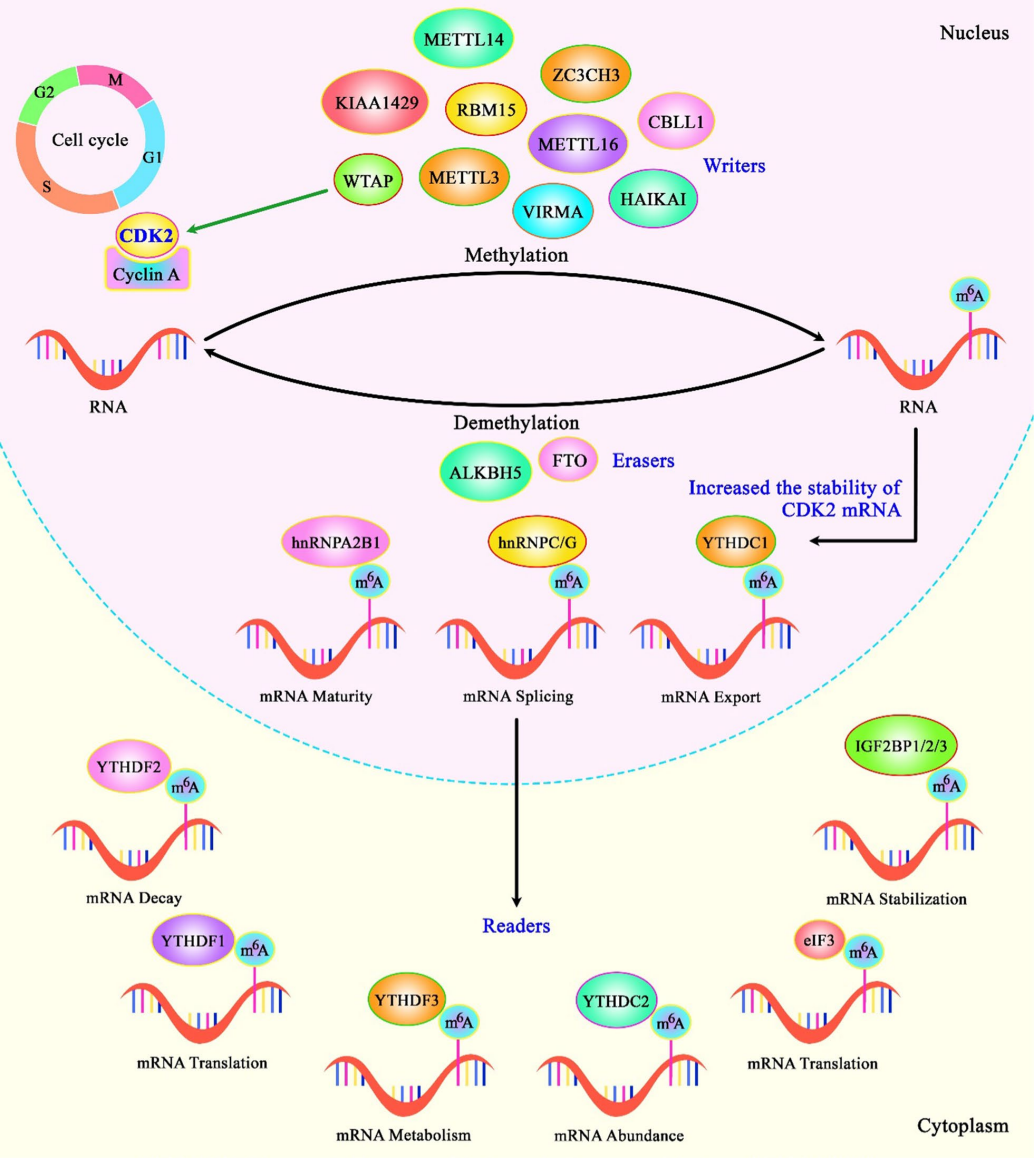
A schematic diagram of CDK1 and the role of WTAP in modulating CDK2 in renal cell carcinoma. Mounting evidence has demonstrated the roles of N6-methyladenosine (m6A) in physiological processes and the progression of various human cancers such as cell cycle regulation that is mostly dependent on cyclins and CDKs. As a component in the m6A ‘writers’, WTAP is detected to be an RNA-binding protein and has a role in the m6A modification, mRNA splicing as well as processing. As an illustration, a recent study has detected that WTAP, an important component of the m6A writer complex, could have an oncogenic role in renal cell carcinoma tumorigenesis via physically binding to CDK2 transcript and promoting its transcript stability [68]

In breast cancer cells, the RNA binding protein KIAA1429 has been shown to interact with CDK1. Although this RNA binding protein is regarded as an N6-methyladenosine-associated regulatory protein, its oncogenic roles in breast cancer are exerted through regulation of CDK1 in an independent manner from its association with N6-methyladenosine (Fig. 1). Treatment of breast cancer cells with 5′-fluorouracil has efficiently reduced expressions of KIAA1429 and CDK1 [10]. Furthermore, siRNA-mediated silencing of CDK1 and CDC20 has significantly repressed cell migration and invasion of two breast cancer cell lines [11]. Another study has shown that knockdown of the ubiquitin-associated domain-containing gene UBAP2L in breast cancer cells suppresses their proliferation, impairs their colony formation aptitude and induces cell cycle arrest at G2/M phase. Most notably, this intervention has led to enhancement of p21 levels, while reducing levels of both CDK1 and Cyclin B1 [12].

Cyclin B/CDK1 has been shown to phosphorylate inhibitor of apoptosis stimulating protein of P53 (iASPP), thus increasing nuclear localization of this protein and its inhibitory effects on p53. In Burkitt lymphoma cells, iASPP has been found to affect activity of transactivation domain p63 (TAp63). In fact, the interplay between CDK1 and iASPP can enhance the suppressive impact of iASPP on p53 and TAp63. Most notably, the tumor suppressor miR-129 has been shown to suppress expression of CDK1 and iASPP through binding with their transcripts. Moreover, CDK1 targeting by miR-129 can lead to inhibition of iASPP phosphorylation, therefore deterring nuclear localization of iASPP and its suppressive impact on p53 and TAp63 [13].

The oncogenic mutation HRAS^V12^ has been found to induce activity of CDK1 and enhance protein *O*-GlcNAcylation, both of them having essential roles in induction of SOX2 expression and cancer stem cell properties in fibroblasts and cancer cell lines harboring *RAS* mutations. Most notably, the CDK inhibitor dinaciclib could reduce the quantities of cancer stem cells originated from these cells [14].

In colorectal cancer cells, knock-down of CDK1 has induced sensitivity to apoptosis. Moreover, CDK1 targeting with a MEK/ERK inhibitor has demonstrated effective impacts on proliferative abilities of these cells [15].

Notably, experiments in the vemurafenib-resistant colon cancer sublines have shown stable activation of CDK1, signifying the role of CDK1 activation in stimulation of resistance to vemurafenib. Adefovir dipivoxil that interrupts the interaction between CDK1 and KCTD12 and induces cell cycle arrest at G2 could inhibit colon cancer cells proliferation and induce sensitivity to vemurafenib [16]. Table 1 shows function of CDKs in cancer cell lines.

**Table 1:** Function of CDK1 based on cell line studies

Tumor type	Targets/ Regulators and Signaling Pathways	Cell line	Function	References
Adrenocortical carcinoma	CENPF	SW13	CENPF/CDK1 signaling pathway was found to regulate the G2/M-phase, thus enhancing progression of adrenocortical carcinoma	[6]
Bladder cancer	PVT1/miR-31/CDK1 axis	RT4, T24, BIU‐87, and 5673	PVT1 facilitated proliferation, migration, and invasion via down-regulating miR-31 to enhance CDK1 expression	[7]
	TFCP2L1	Murine R1, E14TG2a, and gcOct4‐GFP ESCs, HBlEpC, J82, T24, 5637, HT1197, HT1376, and RT4	CDK1-mediated TFCP2L1 phosphorylation was found to have essential role in bladder cancer	[8]
	Cdc25C, Chk1, CDK1-cyclin B1 complex, Myt1, Wee1, phospho-Cdc25C (Ser216), Gadd45α, and 14-3-3 proteins	SCaBER, 5637, T24, UMUC3, TCCSUP, SV-HUC, T24, T24T, TCCSUP, UMUC1, and SV-HUC	Protein kinase D inhibitor “CRT0066101” suppressed expression of Cdc25C, which activates CDK1, but activated Chk1, that inhibits CDK1 and indirectly reduced the CDK1-cyclin B1 complex activity, so it inhibited bladder cancer growth by blocking cell cycle at G2/M	[9]
Breast cancer	KIAA1429	MCF-7, BT474, SUM1315, MDA-MB-231 and MCF-10A	KIAA1429 was found to positively regulate CDK1	[10]
	_	MCF-7 and MDA-MB-231	∆ CDK1: ↓ migration and invasion	[11]
	UBAP2L	MCF-7, ZR-75-30, BT-474, T-47D and MDA-MB-468, and MCF-10A	∆ UBAP2L: ↓ proliferation, colony formation, CDK1 levels, and ↑ cell cycle arrest	[12]
	miR-424	MDA-MB-231, HCC1937, MCF-10A, and HEK-293 T	↑↑ miR-424: ↓ proliferation and ↑ cell cycle arrest via targeting CDK1	[17]
	NUSAP1, and DLGAP5	MCF-7	∆ NUSAP1: ↓ proliferation, migration, and invasion via regulating CDK1 and DLGAP5 expression and ↑ sensitivity to E-ADM	[18]
	RBM7	SUM-1315, MCF-7, BT474, ZR-75-1, and MDA-MB-231	RBM7 was found to bind to the 3'-UTR of CDK1 transcript, which is involved in the stability of CDK1 mRNA RBM7 plays its oncogenic role by increasing the levels of CDK1	[19]
Burkitt lymphoma	miR-129 and iASPP	Raji and CA46	miR-129 was found to target CDK1, so it is involved in inhibiting iASPP phosphorylation and reducing proliferation ∆ CDK1: ↓ iASPP S84/S113 phosphorylation, so blocked iASPP nucleus localization	[13]
Cancer stem cells	RAS/MAPK/CDK1 pathway, SOX2	p53 − / − MEFs, HRASV12-expressing p53 − / − MEF, TIG-3, and TIG-3–SMR, HCT116, SW480, DLD1, HCC827, and H460	RAS/MAPK/CDK1 pathway induces enhanced O-GlcNAc modification and is required for expression of SOX2 and cancer stem cells generation	[14]
	miR-143-3p and miR-495-3p	HcerEpic, C4-1, HeLa, SiHa, and Caski	CDK1 was a target of miR-143-3p and miR-495-3p ↑↑ miR-143-3p or miR-495-3p: ↓ proliferation, migration, invasion, viability and ↑ apoptosis	[20]
	NCK1-AS1/miR-6857/CDK1 axis	CerEpiC, HeLa, C33A and SiHa and CaSki	∆ NCK1-AS1: ↓ proliferation and invasion, and ↑ cell cycle arrest NCK1-AS1 was found to sponge miR-6857, so regulate CDK1/6 protein translation	[21]
Cholangiocarcinoma	_	CCKS-1, TFK-1 and HUCCT-1	∆ CDK1: ↓ proliferation and invasion, and ↑ cell cycle arrest	[22]
	PSMC2	HUCCT1, QBC939, RBE, and HCCC-9810	∆ PSMC2: ↓ proliferation, cell migration, ↑ cell cycle arrest, and apoptosis PSMC2 eas found to regulate its role via regulating CDK1	[23]
Colorectal cancer	KCTD12	HCT116 and HT29	Adefovir dipivoxil: ↓ proliferation, tumorigenesis, and ↑ G2 phase arrest via disrupting the CDK1-KCTD12 interaction ↑↑ CDK1: ↑ vemurafenib resistance	[16]
	MEK/ERK pathway	HT-29, RKO, VACO432, WiDr, DLD1, SW620, DiFi, A375, A19, T29 and VACO432, VT1, NB7	∆ CDK1: ↑ sensitivity to apoptosis A MEK/ERK inhibitor targeting CDK1 has effective role in reduction of cell proliferation	[15]
	miR-378a-5p	SW480, HCT116, SW620, HT-29 and NCM460	CDK1 was a target of miR-378a-5p ↑↑ miR-378a-5p: ↓ proliferation and migration ↑↑ CDK1: ↑ proliferation and migration	[24]
	DPP3	DLD-1, SW480, HCT 116, and RKO	∆ CDK1: ↓ inhibitory effects of DPP3 knockdown ∆ DPP3: ↓ proliferation, migration, ↑ apoptosis and cell cycle arrest DPP3 was found to regulate CRC via CDK1	[25]
	SNHG4/ miR-590-3p/CDK1 axis	FHC, HCT8, LoVo, HCT116, SW620, and HT29	∆ SNHG4: ↓ proliferation, viability, metastasis, and colony formation via targeting miR-590-3p and regulating CDK1	[26]
	NFE2L3, DUX4	HCT116 and HT29	∆ NFE2L3: ↑ levels of DUX4, which is an inhibitor of CDK1	[27]
	SNRPA1	SW480, RKO, HT-29, HCT116, and HEK293T	∆ SNRPA1: ↓ proliferation, ↑ apoptosis SNPRA1 was found to regulate CDK1 in CRC	[28]
Endometrial carcinoma	miR-1271	ECC-1, RL95-2, AN3 CA, and T-HESC	↑↑ miR-1271: ↓ cell proliferation, ↑ apoptosis via targeting CDK1	[29]
Esophageal squamous cell carcinoma	FAM135B, PI3K/Akt/mTOR signaling pathway	KYSE150, ECA109, TE-13, TE-10 and TE-1	∆ FAM135B: ↓ colony formation and ↓ cell cycle protein expression (pP53, CDK1), ↑ cell cycle arrest and ↑ radiosensitivity through regulating PI3K/Akt/mTOR	[30]
Gastric cancer	CASC11 and miR-340-5p	GES-1, MKN7, KATOIII and AZ521	∆ CASC11: ↓ proliferation, ↑ apoptosis and cell cycle arrest CASC11 regulated CDK1 via targeting miR-340-5p	[31]
	ESRRA, CDC25C-CDK1-Cyclin B1 pathway	HGC27, BGC823, MGC803, SGC7901 and GES-1	∆ ESRRA: ↓ cell viability, proliferation, migration, and invasion, EMT process, and ↑ apoptosis ESRRA/DSN1/CDC25C-CDK1-Cyclin B1 pathway was involved in in GC development	[32]
	CDCA5	MGC-803, SGC-7901, and BGC-823	∆ CDK1: ↓ proliferation, colon formation, migration, and invasion CDK1 and CDCA5 were co-expressed in GC cells	[33]
	ISL1	BGC823, MGC803, MKN28, and GES1	is CDK1 phosphorylated ISL1 at serine 269, thus promoted proliferation	[34]
Glioblastoma	p50, BCL-3, NF-κB	U87, A172, T98, U251, and GBM34	CDK1 was found to be up-regulated by temozolomide in an NF-κB related manner ∆ CDK1: ↑ sensitivity cells to temozolomide	[35]
Glioma	FOXD2-AS1/miR-31/CDK1 axis	SVG p12, T98, LN229, U87, U251, and 293FT	∆ FOXD2-AS1: ↓ proliferation, and ↑ cell cycle arrest FOXD2-AS1 was found to sponge miR-31, so regulated CDK1 levels	[36]
Hepatocellular carcinoma	PDK1/β-Catenin	MHCC97H (97H), LO2 and 97H liver cancer stem cells	∆ CDK1/PDK1/β-Catenin: ↓ EMT process RO3306 and sorafenib combination: ↓ 97H CSC growth	[37]
	DEPDC1B	HEP3B2.1-7, SK-HEP-1, huh-7, and HCCLM3	∆ DEPDC1B: ↓ proliferation, migration, colony formation, and ↑ G2 phase arrest, and cell apoptosis The function of DEPDC1B was found to be mediated by CDK1	[38]
	miR-1271-5p	SMMC-7721 and HuH-7	↑↑ miR-1271-5p: ↓ proliferation and ↑ radiosensitivity via targeting CDK1	[39]
	CDK1-PLK1/SGOL2/ANLN pathway	SK-Hep1	∆ CDK1: ↓ expression of PLK1, ANLN, and SGOL2 and resulted in a disordered cell cycle	[40]
	Upf1/SNORD52/CDK1 pathway	Huh7, HepG2, Hep3B, SK-Hep1, HCCLM9, HCCLM3, and HL-7702	∆ SNORD52: ↓ ↓ migration and invasion, and ↑ cell cycle arrest SNORD52 was found to regulate CDK1 by increasing the stability of CDK1 proteins	[41]
Leukemia	PLK1, Aurora B, and TRF1	HL-60	∆ CDK1: ↓ proliferation, ↑ cell cycle arrest via reducing the phosphorylation of PLK1 and Aurora B and negatively regulating TRF1	[42]
Lung cancer	Sox2	A549 and NCI-H520	∆ CDK1: ↑ chemotherapeutic sensitivity CDK1/Sox2 axis was found to regulate the stemness	[43]
	CASC11, miR-302	A547, H157, SPC-A-1 and 16HBE	∆ CASC11: ↓ proliferation via targeting miR-302 and regulating CDK1	[44]
	miR-34c-3p	A549, CALU-1, and HCC827	↑↑ miR-34c-3p: ↓ proliferation, ↑ apoptosis and in KRASmut cells via targeting CDK1	[45]
	NCK1-AS1	A549, NCI-H1299, PC-9 and NCI-H1650	∆ NCK1-AS1 (which regulated CDK1): ↓ proliferation	[46]
	miR-186	A549, H1299, H460, and BEAS-2	Lycorine treatment: ↑ levels of miR-186 and ↓ levels of CDK1: ↓ proliferation and ↑ apoptosis CDK1 was a target of miR-186	[47]
	GP130/STAT3 signaling pathway	A549, 1792, and HEK293T	↑↑ Iron-dependent CDK1 activity: ↑ activaty of the GP130/STAT3 signaling	[48]
	TMPO-AS1 and miR-143-3p	16HBE, H1299, A549, 95D, and H125	∆ TMPO-AS1: ↓ cell viability, ↑ apoptosis TMPO-AS1 regulated CDK1 via targeting miR-143-3p	[49]
	miR-181a	16HBE,, H1299, and A549	↑↑ miR-181a: ↓ proliferation, colony formation, and invasion	[50]
	miR-143 and miR-506	HFL-1, A549, H358, H69-AR, H358, H1975, and Calu-3	↑↑ miR-143 and miR-506: ↓ cell growth via targeting CDK1 and CDK4	[51]
	miR-143 and miR-506	A549, HUVECs	↑↑ miR-143 and miR-506: ↓ angiogenesis, and ↑ cell cycle arrest via targeting CDK1, 4/6 genes, respectively	[52]
Melanoma	Sox2	1205Lu, WM239A, A375, and HCT116	CDK1 was found to be a new regulator of Sox2, so had tumor-initiating capacity in melanoma	[53]
	CHPF	A375	CHPF was found to play its oncogenic role by regulating of CDK1 in malignant melanoma	[54]
Myeloid leukemia	EZH2 and DNMT3A	NIH3T3, 293T, and OCI-AML3	↑↑ DNMT3A mutation-induced CDK1: ↑ proliferation and ↓ apoptosis via modulating the interaction between EZH2 and DNMT3A	[55]
Nasopharyngeal carcinoma	cyclin B1	5-8F and 6-10B NPC	Proteasome inhibitors were found to participate in the accretion of CDK1/cyclin B1, so decreased paclitaxel-induced cell death	[56]
	CDC25C/CDK1/Cyclin B1 pathway	CNE1 and CNE2	appropriate dose of tetrandrine and irradiation treatment: ↓ phosphorylation of CDK1 and CDC25C and ↑ expression of cyclin B1, ↑ cell cycle arrest	[57]
	miR-195-3p	5–8 F, 6–10B, CNE1, CNE2, C666-1, and NP69	↑↑ miR-195-3p: ↑ radiosensitivity via targeting CDK1	[58]
Ovarian cancer	UBE2C	KOV3, A2780, SKOV3/DDP, and A2780/ DDP	∆ UBE2C: ↓ proliferation, cisplatin resistance, and ↑ apoptosis via downregulating CDK1	[59]
	Chk1-CDC25C and P53-P21WAF1 signaling pathway	SK-OV-3 and OVCAR-3	∆ CDK1: ↓ proliferation, ↑ cell cycle arrest, and cell apoptosis	[60]
	TONSL-AS1 and miR-490-3p	OVCAR3 OEC cell line	↑↑ TONSL-AS1: ↑ proliferation via targeting miR-490-3p and regulating CDK1	[61]
	DLEU1/miR-490-3p/CDK1 axis	OVCAR3 and A2780	↑↑ DLEU1: ↑ proliferation, migration, and invasion, and ↓ apoptosis DLEU1 was found to sponge miR-490-3p, so regulate CDK1	[62]
Pancreatic cancer	KRas	MiaPaCa2, Panc1, L3.6pl, A549, A427, H460, Calu6, SW620, DLD1, HCT8	AT7519, (a CDK1, 2, 7, and 9 inhibitor) induces apoptosis CDK hyperactivation was linked with mt KRas dependency	[63]
	miR-143 and miR-506	Panc-1 and MIA-PaCa-2	↑↑ miR-143 and miR-506: ↓ cell growth via targeting CDK1 and CDK4	[51]
Pancreatic ductal adenocarcinoma	_	PATU-T, Hs766T, and HPAF-II	Oxadiazole-based topsentin derivative (compound 6b): ↓ CDK1 expression, and ↑ apoptosis	[64]
	_	different cell lines	Inaciclib was found to be an immune checkpoint inhibitor ∆ CDK1/2/5: ↓ UN-dependent STAT1 expression and activation, ↑ caspase-dependent apoptosis and histone-dependent ICD	[65]
Prostate cancer	TPX2, ERK/GSK3β/SNAIL signaling pathway	BPH-1, LNCaP, C4-2, PC-3, 22RV1	∆ TPX2: ↓ cell activity and migration, EMT process, ↓ expression of CDK1, ↓ the phosphorylation of ERK/GSK3β/SNAIL	[66]
	ABCC5	C4-2, VCaP, ENZ-R, C4-2 and 22Rv1	↑↑ ABCC5: ↑ progression of cancer and resistance to Enzalutamide via the CDK1-mediated phosphorylation of AR ABCC5 was found to inhibit ubiquitination of CDK1 via binding to CDK1 ∆ CDK1: ↑ sensitivity to enzalutamide	[67]

∆ knock-down or deletion, *ICD* immunogenic cell death, *EMT* epithelial-mesenchymal transition, *GC* Gastric cancer, *CRC* Colorectal cancer

### Animal studies

In vivo assessments have shown that down-regulation of miR-31 enhances expression of CDK1 at transcript and protein levels. Down-regulation of PVT1 (an lncRNA which increases expression of CDK1) has led to lessening of bladder tumor size, decrease in the proliferation rate of tumor cells and reduction of CDK1 and Ki-67-expressing cells as demonstrated by immunohistochemistry [7]. In animal models of breast cancer, up-regulation of RBM7 which induces activity of CDK1 has been shown to increase tumor growth [19]. In colorectal cancer, high levels of miR-378a-5p reduces tumor burden through decreasing expression of CDK1 [24]. Moreover, disruption of the interaction between CDK1 and KCTD12 using Adefovir dipivoxil has been shown to reduce in vivo tumorigenesis of colon cancer cells and induce vemurafenib sensitivity in xenografts [16].

Most notably, in animal models of hepatocellular carcinoma, administration of a CDK1 inhibitor along with sorafenib has enhanced the effectiveness of sorafenib [37]. Moreover, in animal models of pancreatic cancer, reduction of phosphorylation of CDK1, 2, 7, and 9 by AT7519 has been associated with reduction of tumor growth [63]. Studies in animal models of other cancers have also verified that decrease in activity of CDKs consistently reduces tumor burden and induces sensitivity to available therapies (Table 2).

**Table 2:** Function of CDK1 in animal models of cancer

Tumor Type	Animal models	Results	References
Bladder cancer	female BALB/c nude mice	∆ PVT1: ↓ tumor volume and tumor weight	[7]
Breast cancer	4–6-week-old female nude BALB/C mice	↑↑ RBM7 (which up-regulates CDK1): ↑ proliferation, tumor growth	[19]
	6-week-old nude mice	∆ KIAA1429 + CDK1: ↓ tumor weight	[10]
Cervical cancer	4–6-week-old BALB/c nude mice	↑↑ miR-143-3p or miR-495-3p: ↓ tumorigenicity	[20]
		∆ NCK1-AS1: ↓ tumor growth and tumor weight	[21]
Cholangiocarcinoma	5-week-old male BALB/cAnNCrj-nu/nu nude mice	∆ CDK1: ↓ tumor growth	[56]
Colorectal cancer	5-week-old male BALB/c mice	dinaciclib and cobimetinib combination: ↓ tumor growth	[15]
	4-week-old male nude mice	↑↑ miR-378a-5p: ↓ tumor growth by targeting CDK1	[24]
	4-week-old female BALB/c nude mice	∆ DPP3: ↓ tumor growth	[25]
	7-week-old BALB/c nude mice	∆ SNHG4 (which regulated CDK1): ↓ tumor growth	[26]
	female BALB/c nude mice	∆ SNRPA1: ↓ tumor formation	[28]
Gastric cancer	4-week-old male nude mice	∆ ESRRA: ↓ tumor growth	[32]
Hepatocellular carcinoma	NOD-SCID mice	Combination of RO3306 (CDK1 inhibitory substance) and sorafenib: ↓ tumor growth and ↓ sorafenib resistance	[37]
	BALB/c nude mice	∆ DEPDC1B: ↓ tumor growth	[38]
	nude mice	↑↑ miR-1271-5p: ↓ tumor growth via targeting CDK1	[39]
	male nude mice	∆ SNORD52: ↓ tumor growth and mass	[41]
Nasopharyngeal carcinoma	5-week-old immunodeficient BALB/c nu/nu female mice	Tetrandrine treatment: ↑ radiosensitivity and ↓ tumor growth	[57]
Ovarian cancer	ovarian xenograft mice	∆ UBE2C: ↓ tumor growth	[59]
	4-week-old female BALB/c mice	↑↑ DLEU1: ↑ tumor growth	[62]
Pancreatic cancer	NOD.Cg-Prkdcscid Il2rgtm1Wjl/SzJ (NSG) mice	AT7519 treatment: ↓ phosphorylation of CDK1, 2, 7, and 9 substrates and ↓ tumor growth	[63]
Pancreatic ductal adenocarcinoma cancer	female C57BL/6 (KPC) or BALB/c (CT26) mic	FNG/dinaciclib combination therapy: ↑ CD8 + T cell-dependent antitumour activity	[65]
Prostate cancer	nude mice	∆ TPX2: ↓ tumor weight	[66]
	4-week-old male BALB/c immunodeficient nude mice	↑↑ ABCC5: ↑ tumor volume and tumor weight	[67]

∆: knock-down or deletion, *GIST* Gastrointestinal stromal tumor

### Investigations in clinical samples

The CDK1-interacting protein CENPF has been found to be over-expressed in human adrenocortical carcinoma samples in correlation with tumor stage and poor overall survival (OS). Further assessment of immune cells infiltration has shown that over-expression of CENPF is associated with different pattern of infiltration of immune cells and high TMB/MSI score. Based on the results of gene-drug interaction assessments inhibitors of this protein, such as Cisplatin, Sunitinib, and Etoposide, can be putative therapeutic modalities for adrenocortical carcinoma [6]. In clinical samples of bladder cancer, activity of the CDK1/TFCP2L1 axis has been found to be associated with aggressive characteristics of tumors including advanced tumor grade, lymphovascular/muscularis-propria invasion, metastatic ability and poor clinical outcomes [8].

Assessment of expression profiles of three breast cancer datasets has led to identification of hub genes that indicate poor prognosis. Further analyses have indicated enrichment of four up-regulated genes, namely CDK1, CDC20, AURKA, and MCM4 in oocyte meiosis and cell cycle pathways. Taken together, bioinformatics methods and experimental validation have suggested these genes as reliable markers for breast cancer [11]. In breast cancer, up-regulation of CDK1 has been associated with short overall, relapse-free and progression-free survival times as well as advanced clinical stage [69]. In patients with cholangiocarcinoma, up-regulation of CDK1 or PSMC2 (which regulates CDK1) has been associated with lymph node metastasis and advanced clinical stage [22] and tumor grade [23], respectively. Table 3 shows the association between dysregulation of CDKs in clinical samples and clinical characteristics.

**Table 3:** Dysregulation of CDK1 in clinical samples

Tumor type	Samples	Expression (Tumor vs. Normal)	Kaplan–Meier analysis (impact of CDK up-regulation)	Association of high expression CDK with clinical data	References
Adrenocortical carcinoma	GEO and TCGA databases	Up-regulation of CENPF (which interacts with CDK1)	Shorter OS	different immune cell populations, and high TMB/MSI score	[6]
Bladder cancer	GEO database 5 bladder cancer tissues and 35 normal tissues	Up-regulation of PVT1 (which regulated CDK1) Up-regulation of CDK1	_	_	[7]
	TCGA dataset	Up-regulation of TFCP2L1	Shorter OS	tumor grade, lymphovascular and muscularis propria invasion, and distant metastasis	[8]
	GEO database (GSE71576) TCGA database: 412 BC patients GEO database (GSE13507: 165 primary bladder cancer samples, 58 ANCTs, 23 recurrent bladder tumor tissues and 10 normal bladder mucosae)	Up-regulation of CDK1	_	tumor grade and recurrence	[70]
Breast cancer	46 PTANCT	Up-regulation of RBM7 (which regulates up-regulation of CDK1)	Shorter OS	_	[19]
	TCGA dataset 72 PTANCT	Up-regulation of KIAA1429	_	advanced clinical stages	[10]
	Oncomine database and GEPIA dataset	Up-regulation of CDK1	Shorter OS, RFP, and PPS	advanced tumor stage	[69]
	GSE42568, GSE45827, and GSE124646 (244 BC tissues and 28 normal breast tissues)	Up-regulation of CDK1	_	_	[11]
	8 PTANCT	Up-regulation of UBAP2L	_	_	[12]
	17 PTANCT	Down-regulation of miR-424	_	_	[17]
	GEO database (GSE21422 and GSE21974)	Up-regulation of NUSAP1 (which regulates CDK1)	_	_	[18]
	GEO database (GSE21422, GSE42568 and GSE45827)	Up-regulation of CDK1	Shorter OS	_	[71]
Cervical cancer	GEO database 60 PTANCT	Up-regulation of CDK1	_	_	[20]
	TCGA dataset (two courts 100 and 120 patients) 31 PTANCT	Up-regulation of NCK1-AS1 (which regulates CDK1)	Shorter OS	_	[21]
Cholangiocarcinoma	54 cholangiocarcinoma patients	Up-regulation of CDK1	Shorter OS	lymph node metastasis and the clinical stage	[22]
	74 CCA tissues and 5 normal tissues	Up-regulation of PSMC2 (which regulates CDK1)	_	advanced tumor grades	[23]
Colorectal cancer	TCGA dataset	Up-regulation of CDK1	_	_	[15]
	TCGA database 22 PTANCT 108 CRC patients	Down-regulation of miR-378a-5p (which targets CDK1) Up-regulation of CDK1	_	tumors in the right colon, lymph node metastasis, and TNM stage	[24]
	99 cancerous tissues and 76 normal tissues	Up-regulation of DPP3	Shorter OS	lymphatic metastases, stage, positive numbers of lymph nodes	[25]
	GSE8671, GSE74602, and TCGA datasets 12 tumor tissues and 12 normal tissues	Up-regulation of SNHG4 (which regulated CDK1)	_	lymphatic or distal metastatic stage	[26]
	GEO database (GSE126092)	Up-regulation of CDK1	Shorter OS	_	[72]
	TCGA database (459 colon cancer samples and 41 normal samples) 5 PTANCT	Up-regulation of NFE2L3	_	_	[27]
	GEO database (GSE21815, GSE106582, and GSE41657)	Up-regulation of CDK1	Shorter OS	gender, tumor type, TNM stage, and KRAS gene mutation	[73]
Endometrial carcinoma	42 PTANCT	Up-regulation of CDK1	_	_	[29]
Esophageal squamous cell carcinoma	151 ESCC tissues and 138 normal esophageal tissues 8 PTANCT 664 ESCC patients and 1733 control tissues	Up-regulation of CDK1	_	_	[74]
Gastric cancer	80 PTANCT GEO database (GSE99416 (6 PTANCT))	Up-regulation of CASC11 (which regulated CDK1)	_	_	[75]
	GEPIA2 database 50 PTANCT 246 patients	Up-regulation of ESRRA	Shorter OS	tumor invasion extent, lymph node/distant metastases and TNM stage	[32]
	Oncomine database, TCGA database and GTEx project (9736 tumor samples and 8726 normal samples)	Up-regulation of CDK1 and CDCA5	_	_	[33]
Glioma	TCGA database (511 low‐grade glioma cases and 156 glioblastoma cases) 30 glioma tissues and 7 normal tissues	Up-regulation of FOXD2-AS1 (which regulated CDK1)	Shorter OS	_	[36]
Hepatocellular carcinoma	3 HCC patients	Up-regulation of CDK1	Shorter OS	_	[37]
	ONCOMINE database AND TCGA, ICGC, and GEO databases	Up-regulation of CDK1	_	immune cell infiltration	[76]
	GEO database (GSE121248, GSE45267 and GSE84402 (132 tumor tissues and 90 normal tissues))	Up-regulation of CDK1	Shorter OS	_	[77]
	178 PTANCT TCGA database	Up-regulation of DEPDC1B (which plays its role via CDK1)	_	pathologic T/N, tumor stage, and gender	[38]
	GEO database (GSE55092, GSE84044 and GSE121248 (119 HBV-related HCC samples and 252 HBV-related non-tumor samples))	Up-regulation of CDK1	_	clinical grading of HCC	[78]
	GEO database (GSE113850) 14 PTANCT	Up-regulation of CDK1	Shorter OS and DFS	HCC occurrence, pathological stages, and survivorship curve	[40]
	GEO database (GSE14520: 225 HCC tissues and 220 normal tissues) TCGA database: 365 patients 59 PTANCT	Up-regulation of CDK1	Shorter OS	_	[79]
	80 PTANCT	Up-regulation of SNORD52 (which regulated CDK1)	Shorter OS and RFS	microvascular invasion and TNM stage	[41]
Hepatocellular carcinoma	GEO database (GSE27635 and GSE28248)	Up-regulation of CDK1	Shorter OS	_	[80]
	GEO database (GSE84402, GSE101685, and GSE112791) TCGA dataset	Up-regulation of CDK1	_	tumor-infiltrate lymphocytes	[81]
Lung cancer	lung tissues	Up-regulation of CDK1	Shorter OS	_	[43]
	GEPIA database (9,736 tumor samples and 8,587 normal controls) 8 PTANC	Up-regulation of CDK1	Shorter OS	advanced tumor stages	[82]
	30 PTANC	Up-regulation of CASC11 (which regulated CDK1)	_	_	[44]
	TCGA database (991 tumor tissues and 91 normal tissues) 14 pairs of KRASmut tumor tissues and ANCTs	Down-regulation of miR-34c-3p (which targets CDK1)	_	_	[45]
	64 PTANC	Up-regulation of NCK1-AS1 (which regulated CDK1)	_	tumor size, TNM stage and lymph node metastasis	[46]
	GEO database (GSE6044 and GSE118370)	Up-regulation of CDK1	Shorter OS and DFS	tumor stages and relative abundance of tumor infiltrating immune cells	[83]
	50 PTANC	Up-regulation of TMPO-AS1 (which regulated CDK1)	Shorter OS	_	[49]
	78 PTANC	Down-regulation of miR-181a	_	histological grade, N status and TNM stage	[50]
	GEO database (5 different microarray datasets: 330 samples)	Up-regulation of CDK1	Shorter OS	_	[84]
Nasopharyngeal carcinoma	99 NPC patients and 46 normal tissues	Down-regulation of miR-195-3p (which sponged CDK1)	_	tumor grade, lymph node metastasis, clinical stage, and radioresistance	[58]
Ovarian cancer	20 tumor tissues and 12 normal tissues	Up-regulation of UBE2C	Shorter OS and PFS	_	[59]
	GEO database (GSE14407, GSE29450, and GSE54388)	Up-regulation of CDK1	Shorter OS	_	[85]
	TCGA dataset 62 PTANC	Up-regulation of TONSL-AS1 (which regulates CDK1)	Shorter OS	_	[61]
	11 benign ovarian tumors, 8 borderline ovarian tumors, 99 ovarian cancer tissues and 15 normal ovary tissues	Up-regulation of DLEU1 (which regulated CDK1) in ovarian cancer tissues	_	differentiation and FIGO staging	[62]
Pancreatic ductal adenocarcinoma	99 PDAC tissues and 71 normal pancreatic tissues	Up-regulation of CDK1	Shorter OS	tumor size and histological grade	[86]
	GEO database (GSE46234, GSE71989, and GSE107610)	Up-regulation of CDK1	Shorter OS and DFS	advanced tumor stage	[87]
Prostate cancer	TCGA database (499 prostate cancer and 52 adjacent tissues)	Up-regulation of TPX2	Shorter OS	high Gleason grade	[66]
	TCGA and GEO databases 149 prostate cancer patients	Up-regulation of ABCC5	Shorter OS and PFS	tumor stage	[67]
Prostate cancer	1,461 patients and 510 normal samples	Down-regulation of miR-205 (which targeted CDK1)	_	bone metastasis	[88]
Rhabdomyosarcoma	GEO database 66 samples (GSE16382 [N = 8] and GSE66533 [N = 58)) and 16 normal striated muscle tissues (GSE39454 [N = 5], GSE17674 [N = 5] and GSE38417 [N = 6])	Up-regulation of CDK1	_	_	[89]
Sebaceous gland carcinoma of the eyelid	3 SGC patients and 1 sebaceous adenoma case	Up-regulation of CDK1 in SGC patients	_	_	[90]
Thyroid cancer	Two tissue microarrays (THC961 and THC1021) (125 cancerous thyroid tissues and 23 non-cancerous thyroid tissues) 46 cancerous thyroid tissues and 64 non-cancerous thyroid tissues 171 cancerous thyroid tissues and 87 non-cancerous thyroid tissues 16 gene microarrays (419 cancerous thyroid tissues and 269 non-cancerous thyroid tissues)	Up-regulation of CDK1	_	_	[91]

*PTANC* pairs of tumor samples and adjacent non-cancerous samples, *OS* Overall survival, *TCGA* Cancer Genome Atlas, *GEO* Gene Expression Omnibus, *HCC* Hepatocellular carcinoma, *TNM* tumor node metastasis, *CRC* Colorectal cancer, *PFS* progression-free survival, *ICGC* International Cancer Genome Consortium, *GEPIA* Gene Expression Profiling Interactive Analysis, *PPS* post-progression survival, and *RFP* recurrence-free probability, *DFS* disease-free survival, *PDAC* Pancreatic ductal adenocarcinoma, *SGC* Sebaceous gland carcinoma, *ESCC* esophageal squamous cell carcinoma, *RFS* recurrence-free survival, *DFS* disease-free survival, *FIGO* International Federation of Gynecology and Obstetrics

## Cyclin-dependent kinase 2 (CDK2)

### Cell line studies

Inactivation of CDK2 has been shown to effectively overcome the differentiation arrest of acute myeloid leukemia (AML) cells. Treatment of AML cells with CDK2-targeted proteolysis-targeting chimeras (PROTACs) has resulted in prompt and effective degradation of CDK2 in various cell lines without similar destruction of other targets. Moreover, this therapeutic agent has induced significant differentiation of AML cells as well as primary patient cells [92]. Another study in AML cells has shown that CDK2 is the only interphase CDK that is degraded through a ubiquitin-dependent proteasomal system. This mode of degradation of CDK2 is associated differentiation of AML cells. KLHL6 has been shown to be the specific E3 ubiquitin ligase which regulates CDK2 degradation. Notably, suppression of CDK2, but not CDK1/4/6, could induce granulocytic differentiation in AML cell lines. From a mechanistical point of view, CDK2 depletion results in reactivation of translation of differentiation pathway. Moreover, the effect of CDK2 in induction of differentiation blockade is exerted through preserving the activity of PRDX2 [93]. Moreover, CDK2 has been shown to down-regulate expression of C/EBPα through ubiquitin-dependent proteasomal degradation system resulting in differentiation blockade in AML. Mechanistically, CDK2-induced C/EBPα down-regulation is facilitated by SKP2. In fact, CDK2 enhances stability of SKP2 through Ser64 phosphorylation leading to C/EBPα ubiquitination. Suppression of CDK2 results in down-regulation of SKP2 and up-regulation of C/EBPα in myeloid cells. Cumulatively, CDK2-SKP2 axis has been identified as a therapeutic target for AML [94]. Another study has shown that GSK8612-mediated TBK1 inhibition and si-TBK1 can regulate CDK2 expression in AML cells through AKT pathway. Suppression of activity of AKT can enhance sensitivity of AML cells to daunorubicin, endorsing the interaction between TBK1 and the AKT/CDK2 axis [95].

Treatment of bladder cancer cells with propofol could inhibit their proliferation and enhance cell apoptosis through regulation of CDK2 expression. Mechanistically, propofol up-regulates expression of a CDK2-targeting miRNA, namely miR-340. Suppression of miR-340 has reversed the impacts of propofol on proliferation and apoptosis of bladder cancer cells. Moreover, suppression of CDK2 can partly reverse the impacts of miR-340 inhibition on proliferation and apoptosis of propofol-treated bladder cancer cells [96].

The Cdk4/6 inhibitor palbociclib has been shown to exert antitumor effects against bladder cancer cells through modification of Cdk2. Palbociclib has been shown to induce apoptosis of bladder cancer cells rather than cell cycle arrest. Activation Cdk2 has an indispensable role in palbociclib-induced apoptosis, as depletion of Cdk2 has suppressed caspase-3 activation and apoptosis. Activation Cdk2 has been shown to induce p-Rad9 mitochondrial translocation and its interaction with Bcl-xl, resulting in Bak activation and induction of apoptosis [97].

**Fig. 2:**
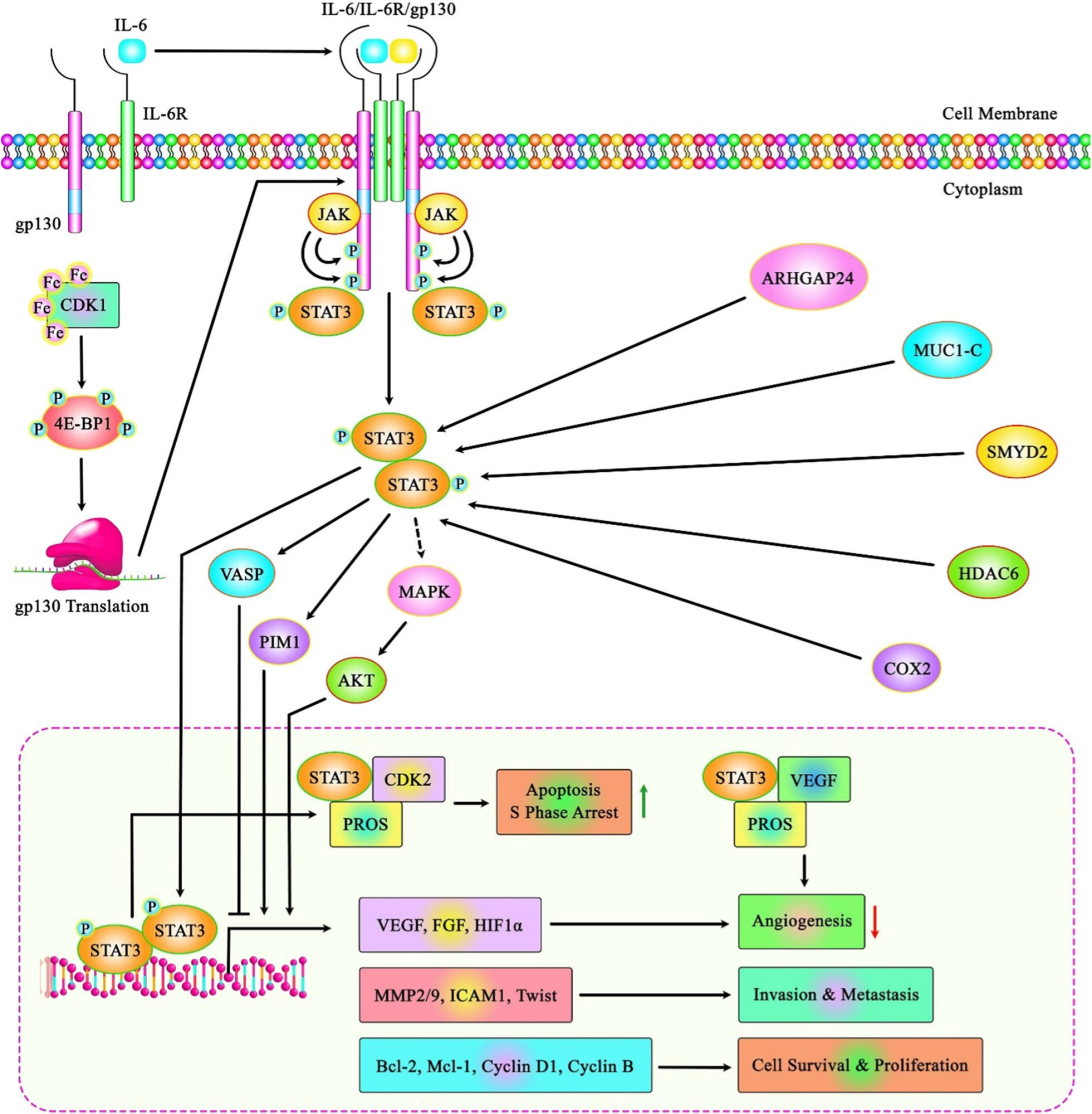
A schematic illustration of the role of STAT3 signaling cascade in regulating CDK1 and CDK2 in lung cancer. Accumulating evidence has illustrated that CDK1/GP130/STAT3 signaling could promote lung cancer tumorigenesis. It has been reported that Iron-dependent CDK1 activity could phosphorylate 4E-BP1, which in turn elevates STAT3 signaling pathway via upregulation of GP130 [48]. Moreover, another research has revealed that PROS could downregulate VEGF induced proliferation, migration, and tube formation in non-small lung cancer cells and inhibits angiogenesis in chorioallantoic membrane assay through attenuating phosphorylation of VEGFR2, Src, and STAT3, thereby inducing sub G1 accumulation, S phase arrest [158]

**Fig. 3:**
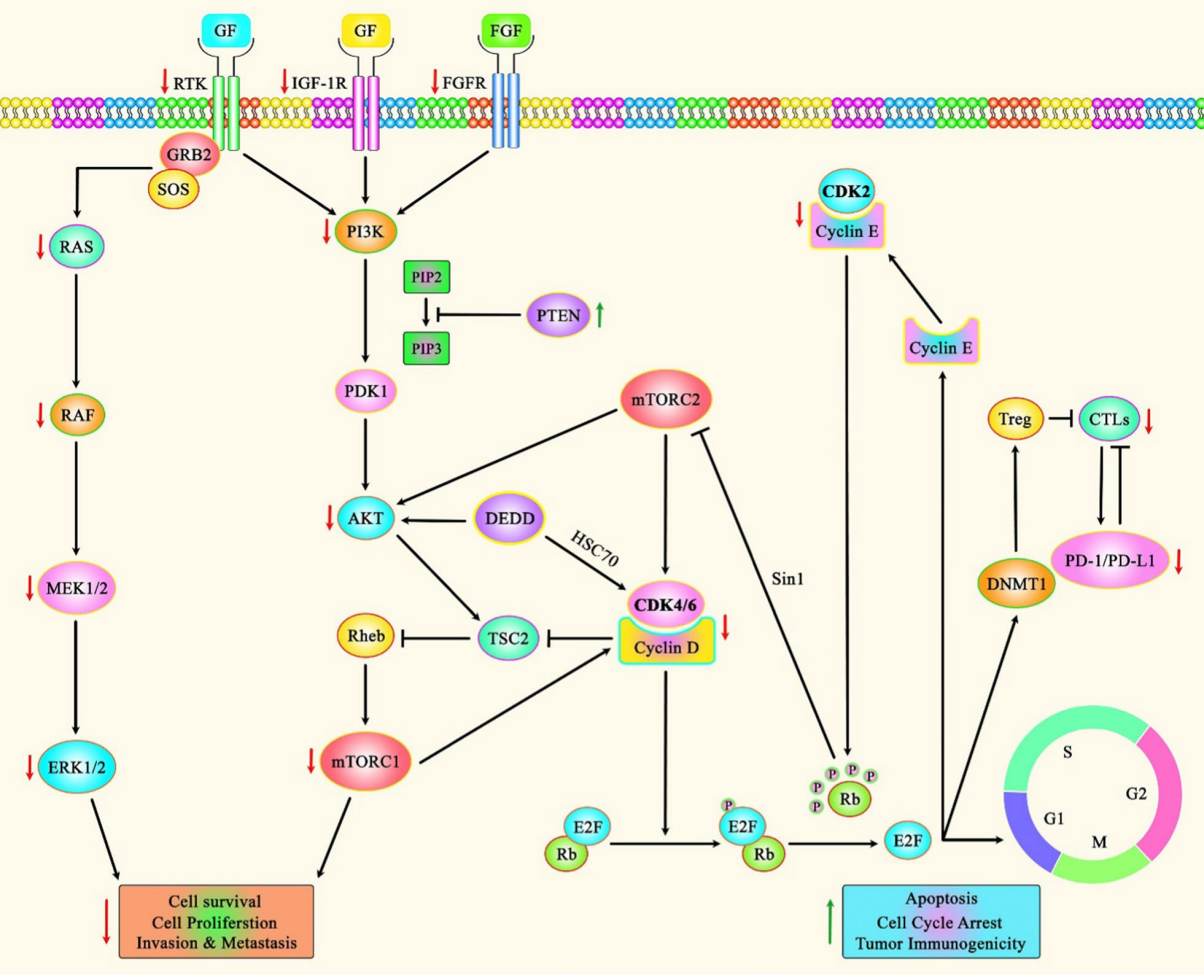
A schematic representation of the role of PI3K/AKT/mTOR and MAPK/ERK signaling pathways in regulating the expression of CDK2 and CDK4/6 in various human cancers

In breast cancer cells, concurrent administartion of CDK2 and CDK4/6 inhibitiors could reverse palbociclib resistance through increasing cell senescence [98]. Another functional study has shown that CDK2-mediated phosphorylation of EZH2 induces and preserves proliferation of triple-negative breast cancer cells [99]. Table 4 summarizes function of CDK2 in different cancer cell lines. Figure 2 illustrates the interaction between STAT3 signaling pathway and CDK1 and CDK2 in lung cancer (Fig. 3).

**Table 4:** Function of CDK2 based on cell line studies

Tumor type	Targets/ Regulators and Signaling Pathways	Cell line	Function	References
Acute myeloid leukemia	CDK2 and CPS2	NB4, U937 and HL60	PROTACs: ↑ CDK2 degradation and ↑ differentiation of AML cell lines CPS2 was found to induce differentiation by CDK2 degradation	[92]
	CDK2-PRDX2 axis, KLHL6	Leu-1-19, NB4, and U937, U2OS, COS-7, HeLa	∆ CDK1: ↑ granulocytic differentiation in AML cell lines and reactivation of differentiation pathway translation KLHL6 was found to mediate degradation of CDK2 CDK2 blocks differentiation in AML cell lines by maintaining the activity of PRDX2	[93]
	CDK2-SKP2 axis and C/EBPα	HL-60, THP-1 and U937	CDK2 enhanced stabilization of SKP2 via phosphorylating it which in turn induced C/EBPα degradation	[94]
	CDK2 and C/EBPα	K562, THP‐1, U937, HEK293T and MCF‐7	CDK2 mediated C/EBPα ubiquitin proteasome degradation leading to destabilization of it which in turn leading to differentiation arrest in AML	[100]
	TBK1 and AKT-CDK2 pathway	Kasumi-1, HL-60 and THP-1	Down-regulation of TBK1 induced daunorubicin sensitivity via the AKT-CDK2 axis GSK8612, a TBK1 inhibitor, reduced TBK1-AKT-CDK2 expression	[95]
	HDAC3-AKT-P21-CDK2 signaling pathway	K562, K562/A02, HL60, HL60/ADR, THP-1, THP-1/ADR, HEK293T,	Chidamide could inhibit HDAC3-AKT-P21-CDK2 signaling so induces sensitivity of anthracycline ∆ HDAC3: ↓ proliferation, ↑ apoptosis, cell cycle arrest at G0/G1 phase, and ↓ AKT, P21, and CDK2	[101]
	CDK2	U937, NB4, HL60, and 293FT	∆ CDK2: ↓ proliferation, ↑ G0 /G 1 phase arrest and sensitivity of AML cells to ATRA-induced cell differentiation	[102]
	CDK2	HL-60	Roscovitine, an inhibitor of CDK2: ↑ ATRA-induced leukemia cell differentiation	[103]
	CDK2, CyclinD3,Hsp90,EGFR, P27, Caspase 7, and TNF	HL-60	Combination of HAA2020 and dinaciclib: ↓ proliferation, survival and ↑ apoptosis via reducing the levels of CDK2, CyclinD3, Hsp90, EGFR, and increasing the levels of P27, Caspase 7, and TNF	[104]
Bladder cancer	miR340/CDK2 axis	5637 cells	Propofol treatment: ↓ proliferation and ↑ apoptosis via regulating miR340/CDK2 axis	[96]
	Cdk2, Rad9 and Bak.Bcl-xl complex	MGC-803, HepG2, NCI-H460, A549, T24 and SKOV-3	Palbociclib: ↑ apoptosis via Cdk2-induced Rad9-mediated reorganization of the Bak.Bcl-xl complex Palbociclib was found to play its role via Cdk2 activation	[97]
	miR-3619, CDK2, β-catenin and p21	5637, EJ, T24, J82 and SV-HUC-1	↑↑ miR-3619: ↓ proliferation, migration, invasion, EMT process and ↑ apoptosis via downregulating β-catenin and CDK2	[105]
	CDK2 and its 5 substrates	T24, J82, and RT4 BC	CDK2 and its 5 substrates was found to be involved in cisplatin chemotherapy	[106]
	MTHFD2, CDK2, and E2F1	HEK‐293T, UMUC3 and T24	MTHFD2 was found to increase CDK2 and induce bladder cancer cell growth by modulating the cell cycle, thus affecting E2F1 activation	[107]
Breast cancer	C-MYC, CDK2, CDK4/6, and cyclin E	MCF7, MCF7-PR, T47D-PR, T47D	∆ CDK2 and CDK4/6: ↓ Palbociclib resistance through inducing senescence	[98]
	CDK2/EZH2 axis and ESR1	T47D, MDA-MB-231 TNBC cells, BT549, Hs578T, SUM-149, and BT 549	Phosphorylation of EZH2 by CDK2 induces tumorigenesis ESR1 gene encoding ERα was found to be a target of CDK2/EZH2 axis ∆ CDK2 or EZH2: ↑ re-expression of ERα and ↑ converting TNBC to luminal ERα-positive	[99]
	TROJAN, CDK4/6, NKRF, RELA, and CDK2	MCF7, T47D and HEK293T	TROJAN induces ER + breast cancer proliferation and CDK4/6 inhibitor resistance via binding to NKRF and suppressing its interaction with RELA, so increases the expression of CDK2	[108]
	BRCA1, cyclin E1, CDK2, PARP	HCC1937, MDA-MB-468, MDA-MB-436, MDA-MB-231, SkBr3, and BT-20	∆ CDK2: ↑ DNA damage to synergize with PARP inhibition	[109]
	ACTL6A/MYC/CDK2 axis	293FT, MCF-7, MDA-MB-468 and MDA-MB-231, ZR-75-1, BT-474, and BT-549, SKBR-3, and SUM159PT	↑↑ ACTL6A: ↑ proliferation via recruitment of MYC and KAT5 on CDK2 promoter, so increasing its levels K03861 (CDK2 inhibitor) and paclitaxel: ↓ growth	[110]
Breast cancer	CDK2 and CDK4	MCF-10A, MDA-MB-231 and Hs578T	4-AAQB treatment: ↑ cell cycle arrest, DNA damage, and apoptosis via suppressing CDK2 and CDK4	[111]
	CDK2	MCF-7	3-hydrazonoindolin-2-one scaffold (HI 5): ↓ proliferation and ↑ G2/M phase arrest via suppressing CDK2	[112]
	MAFG-AS1/ miR-339-5p/CDK2 axis and ER pathway	MCF-7	↑↑ MAFG-AS1: ↑ ER + breast cancer proliferation by sponging miR-339-5p, and in turn increasing CDK2	[113]
	RHBDD1, Akt and CDK2	MDA-MB-231 and MCF7	∆ RHBDD1: ↓ proliferation, migration, invasion, and ↑ apoptosis by suppressing Akt activation and decreasing CDK2 protein level via proteasome pathway	[114]
	p27 Y88, cdk4 and cdk2	MCF7	ALT blocks p27 Y88 phosphorylation and suppresses activity of cdk4 and cdk2	[115]
	Lnc712/HSP90/Cdc37 complex and CDK2	MCF-10A, MDA-MB-231 and MCF-7 and MCF-7/ADM	Lnc712/HSP90/Cdc37 complex increased proliferation via CDK2 activation	[116]
	p27 pY88, cdk4 and cdk2	MCF7, MB231, T47D HCC1954	ALT + PD combination: ↑ cellular senescence and cell cycle arrest via inhibiting both cdk4 and cdk2 (ALT was found to prevent p27 pY88 and inhibit both cdk4 and cdk2)	[117]
	CDK2	MDA-MB-468	Benzamide derivative compound 25: ↓ proliferation, ↑ apoptosis, cell cycle arrest via inhibiting CDK2	[118]
	CDK2	MCF-7	thiazolone and the fused thiazolthione derivatives: ↑ G1/G2-M phase arrest and apoptosis via inhibiting CDK2	[119]
	CDK2, AKT	SKBr3 and T47D	Higenamine: ↑ antitumor effects of cucurbitacin B via suppressing the interaction of AKT and CDK2	[120]
	CDK2	MDA-MB-231, MDA-MB-468	CRIF1-CDK2 interface inhibitors, F1142-3225 and F0922-0913, and Paclitaxel combination: ↓ proliferation, ↑ apoptosis	[121]
	CDK2, pS294, ER	MCF7	CDK2 was found to mediate pS294 formation Selective CDK2 inhibitors suppress pS294 and ER-dependent gene expression ESR1 mutations increased ligand-independent and tamoxifen-resistant tumor growth CDK2-selective inhibitors like Dinaciclib could prevent pS294 formation and suppress ER-dependent gene expression	[122]
	CDK2, PPM1H, p27	MDA-MB-231	↑↑ PPM1H: ↑ paclitaxel sensitivity via dephosphorylation of p27 CDK2 was found to induce resistance to paclitaxel	[123]
Breast cancer	CDK2, CDK9	MDA-MB-23, MDA-MB-436, and Hs578T	CDK2/9 inhibitors, CYC065 and eribulin combination: ↓ proliferation, ↑ apoptosis	[124]
	CDK2, cyclin D1, cyclin E	MCF-7	HSYB, an isomer of HSYA with antioxidative effects: ↓ proliferation and ↑ cell cycle arrest at the S phase via downregulating cyclin D1, cyclin E, and CDK2	[125]
	CDK2	MCF-7	Arylazopyrazole, 8b: ↑ apoptosis and cell cycle arrest The binding mode of 8b was was found to bind to the active site of CDK2 via three hydrogen bonds	[126]
	CDK2, p21	DA-MB-231 and MCF- 7, and HAECs	pyrvinium pamoate and tigecycline combination: ↓ proliferation, levels of CDK2 but ↑ cell cycle arrest at G1/s phase, and levels of p21 increased	[127]
Cervical cancer	hsa_circ_0000520/ miR-1296/CDK2 axis	SiHa, HT-3, Hela, SW756 and ME-180	∆ hsa_circ_0000520: ↓ proliferation and ↑ apoptosis via up-regulating CDK2	[128]
	circ_0084927/miR-1179/CDK2 axis	HeLa, CaSki, SW756 and C-33A, and HcerEpic	∆ circ_0084927: ↓ proliferation and ↑ cell cycle arrest via regulating miR-1179/CDK2 axis	[129]
	circZFR, SSBP1, CDK2/cyclin E1 complexes, p-Rb, and E2F1	HeLa and SiHa	∆ circZFR: ↓ proliferation, migration, invasion, and tumor growth circZFR interacted with SSBP1, so promotied the assembly of CDK2/cyclin E1 complexes, and induced p-Rb phosphorylation	[130]
	CDK2/E1complex	HeLa	Thiazol-hydrazono-coumarin hybrids, compound 8a, led to cell cycle attesst at G0/G1 phase and apoptosis by targeting CDK2/E1complex	[131]
Cholangiocarcinoma	CDK2/5/9	HuCCT1 and KMCH	Dinaciclib treatment: ↓ proliferation and ↑ apoptosis via suppressing CDK2/5/9	[132]
Colorectal cancer	NPTX1, cyclin A2, CDK2, and Rb-E2F signaling	SW480 and HCT116	↑↑ NPTX1: ↓ proliferation via downregulating cyclin A2 and CDK2, thereby regulating the Rb-E2F signaling	[133]
	CDK2	HCT116	Topane-based compounds (Compounds 26 and 33) could be anticancer agents via inhibiting CDK2 inhibitors	[134]
	MEX3A and CDK2	HIEC-6, SW480, HCT116 and HT29	∆ MEX3A: ↓ viability, proliferation and invasion and ↑ apoptosis via downregulating CDK2	[135]
	CDK2/9	CRC057, CRC119, CRC16-159, CRC240, CRC247, and CRC401	Dual CDK2/9 inhibition: ↑ G2-M arrest and anaphase catastrophe	[136]
	SLCO4A1-AS1, Cdk2, c-Myc	HT29, LoVo, HCT116, SW620, and SW480, and NCM460	SLCO4A1-AS1 promotes colorectal tumorigenicity by increasing Cdk2 levels and activating the c-Myc signaling	[137]
Gastric cancer	CDK2/SIRT5 axis	MGC‐803 and SCG‐7901	∆ CDK2: ↓ aerobic glycolytic capacity and ↑ levels of the SIRT5 tumor suppressor	[138]
	LINC01021, CDK2, CDX2, KISS1	SGC-7901, NCI-N87, BGC-823, and GES1	∆ LINC01021: ↓ migration, invasion, and angiogenesis via inducing the binding between CDX2 and KISS1, and suppressing that between CDK2 and CDX2	[139]
	PCBP2 and CDK2	HGC‐27 and MKN‐45	∆ PCBP2: ↓ Colony formation and viability	[140]
Glioblastoma	Cyclin-CDK2 Pathway	GBM8901 and U87	Water extract of G. lucidum: ↓ proliferation, migration, and ↑ mitochondria-mediated apoptosis and cell cycle arrest at S phase via the cyclin-CDK2 pathway	[141]
Glioma	LINC00958/ miR-203/CDK2 axis	SHG44, U87, U251, A172, and NHAs	∆ LINC00958: ↓ proliferation, invasion, and ↑ cycle arrest at G0/G1 phase LINC00958 promotes gliomagenesis via miR-203/CDK2 axis	[142]
	HSP90AA1-IT1/miR-885-5p/CDK2 axis	NHA, U87MG and U251	∆ HSP90AA1-IT1: ↓ viability, proliferation, EMT, invasion and migration and ↑ apoptosis HSP90AA1-IT1 plays its role via regulating miR-885-5p/CDK2 axis	[143]
Hepatocellular carcinoma	CDK2/4/6, cyclin D/E, Rb	QGY7703 and Huh7	vanoxerine dihydrochloride treatment: ↑ G1-arrest, apoptosis, and ↓ expressions of CDK2/4/6	[144]
	HNRNPU, CDK2	HEK293T, HepG2 and Huh7, MHCC97H	↑↑ HNRNPU: ↑ proliferation via enhancing the transcription of CDK2	[145]
	EGFR-CDK2 signaling	human hepatoma cells	It was found that Cinobufagin could play its antitumor effects by suppressing EGFR-CDK2 signaling	[146]
	MAPRE1 and CDK2	Huh7	MAPRE1 was found to bind with CDK2 and promote HCC progression	[147]
	OLA1, P21, and CDK2	Hep3b, Hep G2, LM3, MHCC-97H and HEK293T	∆ OLA1: ↓ proliferation, migration, invasion, and G0/G1 ↑ phase arrest and apoptosis OLA1 promotes tumorigenicity via binding with P21 and up-regulating CDK2 expression	[148]
	TPT1-AS1, CDK2	SNU-398 and SU.86.86	↑↑ TPT1-AS1: ↓ proliferation via down-regulating CDK2	[149]
	LINC00630, E2F1, CDK2	Bel-7402, SK-Hep1, MHCC-97H, HepG2, and L02	↑↑ LINC00630: ↑ proliferation and ↓ apoptosis via enhancing the binding of E2F1 to the CDK2 promoter region, so promoting CDK2 transcription	[150]
Leukemia	CDK2, p21, p27, p53 and FasR	THP-1 and NHMs	Combination of DOX and PGZ: ↓ cell growth and ↑ G2/M arrest via reducing the levels of CDK2 and increasing the levels of p21, p27, p53 and FasR	[151]
	CDK2	MOLT-4 and HL-60	Pyrazolo[1,5-a]pyrimidines (5 h and 5i) showed the best CDK2 inhibitory activity	[152]
Liver cancer	miR-155, H3F3A CDK2, P21WAF1/CIP1	Hep3B	miR-155 inhibits H3F3A, so promotes the phosphorylation modification of CDK2, thus, miR-155 suppresses the transcription and translation of P21WAF1/CIP1	[153]
Lung cancer	miR-597/CDK2 axis	H1299 and PC-9	↑↑ miR-597: ↓ proliferation via targeting CDK2	[154]
	p21/CDK2/Rb signaling pathway	NSCLC cells	PPI was found to disturb CDK2 function through increasing p21, thus PPI could suppress Rb via the p21/CDK2/Rb signaling pathway PPI and Palb combination: ↑ anti-cancer ability on NSCLC	[155]
	CCNA2-CDK2 complex and AURKA/PLK1 pathway	A549 and NCI-H1975, BEAS-2B, and LLC	Tanshinone IIA: ↓ cancer progression via regulating CCNA2-CDK2 complex and AURKA/PLK1 pathway	[156]
	CDK2/9	ED1, LKR13, 393P, H522, H1703, A549, Hop62, and H2122	CDK2/9 inhibitor, CCT68127: ↓ growth, and ↑ G1 or G2/M arrest	[157]
	STAT3/ VEGF/ CDK2 axis	A549 and H460	PROS plays its antiangiogenic role via inhibiting STAT3/ VEGF/ CDK2 axis	[158]
	AKT, CDK2	A549, A427, NCI-H23, NCI-H358, NCI-H1975, and NCI-H1650	A-674563, a putative AKT1 inhibitor that altered cell cycle progression and off-target CDK2 inhibition, suppresses tumor growth more effectively than the pan-AKT inhibitor, MK-2206	[159]
Medulloblastoma	CDK2 and MYC	MYCN-driven mouse MB cells and hindbrain NSCs, Sai2, AF22, MB002, CHLA25, Kelly	BET bromodomain inhibition and CDK2 inhibition: ↑ cell cycle arrest and apoptosis via suppressing MYC expression and MYC stabilization	[160]
Melanoma	CDK2	MDA-MB-435 and SNB-75, WI-38	Quinazolinone-based derivatives (compounds 5c and 8a) had significant growth inhibition against melanoma via inhibiting CDK2	[161]
Melanoma and non-melanoma skin cancers	CDK2	A375 and SK-Mel-28, A431 and UWBCC1	Flavonol-based derivatives of fisetin, compounds F20, F9 and F17, were found as c-Kit, CDK2 and mTOR inhibitors	[162]
Neuroblastoma	CDK2, MDM2, CDK1, PSMD14 and TSPO (p53 signaling pathway)	IMR32	Down-regulation of CDK2 showed that MDM2, CDK1, PSMD14 and TSPO could be key target genes of CDK2	[163]
Ovarian cancer	CDK2, EZH2, ESR1	SKOV3, OVCA433, CAOV3, DOV13, A2780, OVCA420	∆ CDK2: ↓ phosphorylation of EZH2 at T416, thus increased the expression of its downstream target ERα gene (ESR1)	[164]
	PLAC2 and CDK2	UWB1.289	↑↑ PLAC2: ↑ proliferation via regulating CDK2	[165]
	Cul4B, miR-372, CDK2 and CyclinD1	Hey, PEA-1, SKOV-3 and OVCAR3	↑↑ Cul4B: ↑ proliferation by sponging miR-372 and regulating CDK2 and CyclinD1	[166]
Prostate cancer	CDK2 and PI3K/Akt pathway	PC-3, DU-145 and 22RV1	∆ CDK2: ↓ invasion and metastasis via inactivating PI3K/Akt pathway	[167]
Renal cell carcinoma	SKP2-p21/p27-CDK2 axis	786-O, 769-P, OSRC-2, Caki-1, and HK-2	Nobiletin: ↓ proliferation and ↑ G1 cell cycle arrest and cell apoptosis via decreasing SKP2 by reducing its transcriptional level, thus increasing p27 and p21 levels, which inhibited CDK2	[168]
	WTAP and CDK2	HK2, Caki-1, Caki-2, ACHN, 769P, 786-O	∆ WTAP: ↓ proliferation WTAP plays its oncogenic role via binding to CDK2 transcript and increasing its transcript stability	[68]
	TSG101, c-myc, cyclin E1 and CDK2	A498 and 786-O	∆ TSG101: ↓ proliferation, colony formation and ↑ G0/G1 arrest via down-regulating c-myc, cyclin E1 and CDK2	[169]
Soft tissue leiomyosarcoma	PLA2G10, cyclin E1 and CDK2	SK-LMS-1	PLA2G10 promotes tumorigenicity via enhancing expression of cyclin E1 and CDK2	[170]
T-cell acute lymphoblastic leukemia	SIRT1, p27, CDK2, SKP2	CCRF-CEM, MOLT4, KG-1, THP-1. MV4–11, K562, U937 and 293T	SIRT1 was found to by deacetylate CDK2 and induce the interaction between p27 and SKP2 leading to phosphorylation of p27, thus the degradation of p27 Notch1/Myc axis increased SIRT1 protein level	[171]

∆ knock-down, deletion or inhibition, *PROTACs* first-in-class CDK2-targeted proteolysis-targeting chimeras, *DOX* Doxorubicin, *PGZ* pioglitazone, *TNBC* Triple-negative breast cancer, *4-AAQB* 4-acetyl-antroquinonol B, *ALT* a splice variant of Brk, *HSYB* Hydroxysafflor yellow B, *Palb* Palbociclib, *PPI* Polyphyllin I)

Recent study has detected that upregulation of PTEN and Rb expression levels could lead to promoting sensitivity to CDK4/6 inhibitors, which could in turn result in reducing the expression of AKT and PI3K in ER-Positive Breast Cancer. Whereas, acquired loss of Rb and PTEN expression could induce resistance to CDK4/6 inhibitors in patients, and thereby promoting hyperactivation of CDK2 and CDK4 [172]. Moreover, other finding points out that IGF1R overexpression, as an escape mechanism, could elevate resistance to CDK4/6 inhibitors in Ewing sarcoma. Therefore, dual targeting of CDK4/6 and IGF1R could play an effective role in providing a candidate synergistic combination for clinical application in this disease and promoting inhibition of the cell cycle as well as PI3K/mTOR axis in tumor cells [173]. In addition, a recent clinical study has revealed that suppression of CDK4/6 phosphorylation and the complex with cyclin D as well as downregulating PI3K/AKT/mTOR signaling cascade could remarkably reduce cell viability, induce apoptosis, and promote the percentage of cells in G1 phase in hepatocellular carcinoma [174]. All the information regarding the role of these cascades involved in the regulation of CDK2 and CDK4/6 expression in various types of human cancers can be seen in Tables 4 and 10.

### Animal studies

Depletion of CDK2 has led to blockade of AML cells growth in animal models and increased survival of xenograft mice models [93]. Another study in animal models of AML has shown that concomitant administration of chidamide and doxorubicin could inhibit HDAC3-AKT-P21-CDK2 signaling and reduce tumor growth [101].

Another experiment in an animal model of bladder cancer has shown the anticancer role of Cdk2 activation in palbociclib-treated animals, indicating that the anticancer effect of palbociclib is exerted via Cdk2 activation [97]. In xenogaft models of breast cancer, depletion of CDK2 and CDK4/6 has reduced tumor growth and palbociclib resistance [98]. Similar results have been reported in animal models of other types of cancers (Table 5).

**Table 5:** Function of CDK2 in animal models of cancer

Tumor Type	Animal models	Results	References
Acute myeloid leukemia	NOD/SCID mice	∆ CDK1: ↓ tumor growth and ↑ survival of AML-bearing mice	[92]
	6–8-week-old NOD/SCID immunodeficient mice	Chidamide combined with doxorubicin could inhibit HDAC3-AKT-P21-CDK2 signaling pathway and reduce tumor growth	[101]
	4–5-week-old female NOD/SCID mice	∆ CDK2 and ATRA combination therapy: ↓ engraftment of leukemia cells and ↑ primary AML blasts differentiation	[102]
	BALB/c or C57BL/6 mice	∆ CDK2: ↓ proliferation and ↑ senescence, thus delayed MYC/BCL-XL-driven AML	[175]
Bladder cancer	Pathogen-free male BALB/C nude mice	Palbociclib was found to play its anticancer role via Cdk2 activation	[97]
	4-week-old male BALB/c-nude mice	↑↑ NPTX1: ↓ tumor volume and weight	[105]
Breast cancer	4-week-old BALB/c nude mice	∆ CDK2 and CDK4/6: ↓ proliferation, growth, and ↓ Palbociclib resistance	[98]
	Female BALB/c mice	combination of either CDK2 or EZH2 inhibitor with tamoxifen: ↓ tumor growth and ↑ survival	[99]
	6-week-old female BALB/c nude mice	∆ TROJAN: ↓ tumor growth and tumor volume	[108]
	female NOD-SCID-IL2γR − / − (NSG) mice	Combination PARP and CDK2 inhibition: ↑ tumor regression and survival	[109]
	5–6-week-old female BALB/c-nu mice	↑↑ ACTL6A: ↑ tumor growth K03861 (CDK2 inhibitor) and paclitaxel: ↓ growth	[110]
	NOD/SCID mice	4-AAQB treatment: ↓ tumor growth via suppressing CDK2 and CDK4	[111]
	female BALB/c mice	∆ Lnc712: ↓ tumor growth Via suppressing CDK2	[116]
	female NOD/SCID mice	ALT + PD combination: ↓ tumor growth via inhibiting both cdk4 and cdk2	[117]
	female BALB/c nude mice	Higenamine and cucurbitacin B: ↓ tumor growth via suppressing the interaction of AKT and CDK2	[120]
	5–6-week old female athymic nu/nu mice	CDK2/9 inhibitors, CYC065 and eribulin combination: ↓ tumor volume	[124]
Cervical cancer	4-week-old BALB/C nude mice	∆ hsa_circ_0000520: ↓ tumor volume and weight	[128]
	4–6-week-old female BALB/c nude mice	∆ circZFR: ↓ tumor growth	[130]
Cholangiocarcinoma	6-week old NSG mice	Dinaciclib and gemcitabine combination: ↓ tumor growth	[132]
Colorectal cancer	nude mice	↑↑ NPTX1: ↓ tumor growth via downregulating CDK2	[133]
	8–10-week-old SCID mice	Dual CDK2/9 inhibition: ↓ tumor growth	[136]
	5-week-old athymic nude BALB/c mice	∆ SLCO4A1-AS1: ↓ tumor growth	[137]
Gastric cancer	4–6-week-old nude BALB/c mice	∆ LINC01021: ↓ tumor volume and weight	[139]
Glioma	6-week-old male BALB/c mice	∆ LINC00958: ↓ tumor growth	[142]
	male BALB/c nude mice	∆ HSP90AA1-IT1: ↓ tumor growth	[143]
Hepatocellular carcinoma	4-week-old female BALB/c-nu, nude mice	∆ HNRNPU: ↓ tumor volume and weight	[145]
	6–8-week-old male BALB/C nude mice	∆ OLA1: ↓ tumor growth and weight	[148]
	Nude mice	↑↑ TPT1-AS1: ↓ tumor growth	[149]
	4-week-old athymic BALB/c mice	↑↑ miR-155: ↑ tumor weight	[153]
Lung cancer	6–8-week-old male immunocompetent 129S2/SVPasCrl mice	CDK2/9 inhibitor, CCT68127: ↓ tumor growth	[157]
	BALB/c athymic nude mice	PROS reduced tumor volumes and weights via inhibiting STAT3/ VEGF/ CDK2 axis	[158]
Medulloblastoma	6–8-week-old female Athymic Nude-Foxn1nu mice	BET bromodomain inhibition and CDK2 inhibition: ↓ tumor growth	[160]
Ovarian cancer	6-week old BALB/nude mice	↑↑ PLAC2: ↑ tumor growth via regulating CDK2	[165]
Renal cell carcinoma	4–6-week-old BALB/c athymic nude mice	nobiletin and palbociclib combination: ↓ tumor growth	[168]
	5-week-old female BALB/c nude mice	∆ WTAP: ↓ tumor growth	[68]
Soft tissue leiomyosarcoma	5-week-old female BALB/c nude mic	∆ PLA2G10: ↓ tumor growth and weight	[170]
T-cell acute lymphoblastic leukemia	8-week-old female C57BL/6J mice	∆ SIRT1: ↑ lifespan of T-ALL model mice	[171]

∆ knock-down, deletion or inhibition, *NOD/SCID* nonobese diabetic/severe combined immunodeficiency, *AML* Acute myeloid leukemia, *NSG* NOD scid gamma, *T-ALL* T-cell acute lymphoblastic leukemia, *SCID* severe combined immunodeficient, *NSG* NOD scid gamma, *T-ALL* T-cell acute lymphoblastic leukemia, *SCID* severe combined immunodeficient)

### Investigations in clinical samples

Up-regulation of CDK2 has been reported in diverse types of cancers. In AML, up-regulation of HDAC3-AKT-P21-CDK2 signaling has been associated with shorter event-free and overall survival (OS) times [101]. In bladder cancer, expression of CDK2 has been increased, while expression of a CDK2-targeting miRNA, namely miR-3619 has been decreased. These observations have been associated with advanced tumor stage and grade [105]. In breast cancer, up-regulation of MTHFD2, which contributes in the cell cycle through binding to CDK2, has been associated with shorter OS, tumor grade and stage [107]. Other studies have shown up-regulation of a number of CDK2-interactiong circRNAs such as hsa_circ_0000520 [128], circ_0084927 [129] and circZFR [130] in cervical cancer patients. Notably, up-regulation of circZFR has been associated with lymphatic metastasis in this type of cancer [130]. Several other studies have found association between dysregulation of CDK2 or its interacting partners and clinical data of patients (Table 6).

**Table 6:** Dysregulation of CDK2 in clinical samples

Tumor type	Samples	Expression (Tumor vs. Normal)	Kaplan–Meier analysis (impact of regulators dysregulation)	Multivariate Cox regression analysis	Association of dysregulation of regulators with clinicopathologic characteristics	References
Acute myeloid leukemia	44 patients with AML and 20 healthy controls	Up-regulation of TBK1 (which regulated CDK2)	_		_	[95]
	27 patients with relapsed/refractory AML with anthracycline resistance TCGA database	Up-regulation of HDAC3-AKT-P21-CDK2 signaling	Shorter OS and EFS		_	[101]
Bladder cancer	GSE32894 TCGA dataset 40 patients	Up-regulation of MTHFD2	Shorter OS	_	grade and stage	[107]
	33 PTANCT	Down-regulation of miR-3619 (which regulated CDK2) Up-regulation of CDK2	Shorter OS	miR-3619 and p21 expressions were found to be an independent risk factors for poor OS	tumor stage and grade	[105]
Breast cancer	344 patients	Up-regulation of ACTL6A/MYC/CDK2 axis	Shorter OS and RFS	High levels of ACTL6A and T, N classification were found as independent prognostic factors for the 5-year OS in TNBC subtype.​	_	[110]
	METABRIC dataset	Up-regulation of CDK2 and CDK4	Shorter OS	_	_	[111]
	116 breast cancer tissues and 39 adjacent normal tissues 84 breast cancer patients	Up-regulation of RHBDD1 (which regulated CDK2)	_	_	pathological tumor (pT) stage, pathological TNM stage and estrogen receptor (ER) expression	[114]
	TCGA dataset	Up-regulation of CDK2	_	_	_	[122]
Cervical cancer	108 patients and 54 normal controls	Up-regulation of Cyclin A and CDK2	Shorter OS	_	_	[176]
	GEO database (GSE102686) 52 PTANCT	Up-regulation of hsa_circ_0000520 (which regulated CDK2)	_	_	_	[128]
	GSE102686	Up-regulation of circ_0084927 (which regulated CDK2)	_	_	_	[129]
	GEO database (GSE102686) 30 PTANCT 10 advanced cervical cancer tissues, and 7 normal cervical tissues TCGA dataset: 306 cervical cancer tissues and 13 healthy cervical tissues	Up-regulation of circZFR	_	_	lymphatic metastasis	[130]
Cholangiocarcinoma	TCGA database	Up-regulation of CDK2/5/9	_	_	_	[132]
Colorectal cancer	TCGA dataset 8 PTANCT	Down-regulation of NPTX1 (which regulated CDK2)	_	_	_	[133]
	TCGA dataset	Up-regulation of MEX3A (which regulated CDK2)	_	_	_	[135]
	109 PTANCT 158 PTANCT TCGA and GSE9348, GSE21510, GSE23878 and GSE33113 datasets	Up-regulation of SLCO4A1-AS1 (which regulated CDK2)	Shorter OS and DFS	SLCO4A1-AS1 expression was found to be an independent risk factor	TNM stage	[137]
Gastric cancer	GEO database (GSE13911: 38 gastric cancer samples and 31 normal samples)	Up-regulation of LINC01021 (which regulated CDK2)	Shorter OS	_	pathological stage, metastasis, differentiation level, and tumor size	[139]
	100 PTANCT	Up-regulation of PCBP2 (which regulated CDK2) Up-regulation of CDK2	_	_	_	[140]
Glioma	TCGA, GTEx, CGGA, CancerSEA, and TISCH databases	Up-regulation of CDK2	Shorter OS	_	Grade, endothelial cells, macrophage, and NK cells	[177]
	35 PTANCT	Up-regulation of LINC00958 (which regulated CDK2)	Shorter OS	_	_	[142]
	113 PTANCT	Up-regulation of HSP90AA1-IT1 (which regulated CDK2)	_	_	pathological grades	[143]
Growth hormone adenomas	46 GHPA patients	Up-regulation of cyclin E and Cdk2	_	_	invasion	[178]
Hepatocellular carcinoma	75 PTANCT	Up-regulation of MINCR and CDK2	Shorter OS	_	tumor size, TNM stage, lymph node metastasis, and serum alpha-fetoprotein levels	[179]
	TCGA dataset TCGA dataset: 371 patients (including 50 PTANCT) from	Up-regulation of HNRNPU (which regulated transcription of CDK2)	Shorter OS	_	advanced tumor stage	[145]
Hepatocellular carcinoma	TCGA dataset (351 tumor CC tissues, 50 normal tissues)	Up-regulation of MAPRE1	Shorter OS,RFS, PFS, DSS	_	_	[147]
	TCGA and GEO databases (GSE6764, GSE29721, GSE45436 and GSE62232) 105 PTANCT	Up-regulation of OLA1 (which regulated CDK2)	Shorter OS and DFS	OLA1 was found to be an independent prognostic factor for OS and DFS	tumor size, PVTT, TNM stage and tumor differentiation degree	[148]
	62 PTANCT	Down-regulation of TPT1-AS1 (which regulated CDK2)	_	_	clinical stages	[149]
	GEPIA database 63 PTANCT	Up-regulation of LINC00630 (which regulated CDK2)	_	_	TNM stage and lymph node metastasis	[150]
Lung cancer	GEO and TCGA databases	Up-regulation of CDK2	_	_	IC50 of 89 antitumor drugs	[180]
	50 PTANCT	Down-regulation of miR-597	Shorter OS	_	pathological stage	[154]
Ovarian cancer	64 PTANCT	Up-regulation of PLAC2 and Cdk2	Shorter OS	_	_	[165]
	4 PTANCT 20 PTANCT	Up-regulation of Cul4B (which regulated CDK2)	Shorter OS and RFS	Tumor grade, Cul4B expression were found to be independent risk factors of patient DFS but while tumor grade, FIGO stage and Cul4B expression were identified as independent risk factors of patient OS	FIGO stage	[166]
Prostate cancer	GEO datasets (GSE6605 and GSE6606)	Up-regulation of CDK2	Shorter OS	_	recurrence	[167]
Renal cell carcinoma	85 PTANCT TCGA dataset	Up-regulation of WTAP (which regulated CDK2)	Shorter OS	_	tumor size and TNM stage	[68]
	15 PTANCT	Up-regulation of TSG101 (which regulated CDK2)	_	_	_	[169]
Soft tissue leiomyosarcoma	TCGA dataset 31 STLMS cases with or 22 cases without relapse after primary therapy	Up-regulation of PLA2G10 (which regulated CDK2)	worse RFS	_	_	[170]

*PTANC* pairs of tumor samples and adjacent non-cancerous samples, *AML* Acute myeloid leukemia, *OS* Overall survival, *EFS* event-free survival, *GHPA* Growth hormone adenomas, *ANCTs* adjacent non-cancerous tissues, *TNM* tumor node metastasis, *TCGA* Cancer Genome Atlas, *GEO* Gene Expression Omnibus, *RFS* recurrence-free survival, *FIGO* International Federation of Gynecology and Obstetrics, *DSS* disease‐specific survival, *PFS* progression-free survival, *RFS* relapse‐free survival, *STLMs* Soft tissue leiomyosarcoma

## Cyclin-dependent kinase 3 (CDK3)

### Cell line studies

CDK3 has been shown to participate in regulation of cell cycle transition at G0/G1 and G1/S phases. Up-regulation of CDK3 in breast cancer cells has suppressed their migration and invasion. Further experiments in these cells have identified miR-4469 as a CDK3-targeting miRNA. Consistent with this finding, miR-4469-induced enhancement of cell motility could be obliterated by CDK3 up-regulation. Assessments of RNA-seq data and western blot assay have indicated inhibition of Wnt pathway by CDK3 expression. Besides, Wnt3a treatment could abolish the inhibitory effect of CDK3 in cell motility, indicating the role of CDK3 as an upstream regulator of Wnt signaling in these cells [181].

CDK3 has also been reported to participate in ERα signaling and resistance to tamoxifen. The anti-cancer agent norcantharidin (NCTD) has been found to regulate miR-873/CDK3 axis. Treatment of breast cancer cells with NCTD has led to reduction of transcriptional activity of ERα but not ERβ via influencing activity of miR-873/CDK3 axis. Moreover, NCTD has been shown to inhibit proliferation of breast cancer cells and induce sensitivity to tamoxifen via this axis. Mechanistically, NCTD blocks tamoxifen induced transcriptional activity and ERα downstream gene expression. Moreover, it reestablishes tamoxifen induced recruitment of ERα co-repressors [182]. The CDK3 targeting miRNA, miR‐125a‐3p has also been revealed to inhibit transactivation of ERα and prevail tamoxifen resistance in ER + breast cancer cells [183]. Similarly, miR-873 has been found to regulate transcriptional activity of ERα and resistance to tamoxifen through influencing expression of CDK3 in breast cancer cells [184].

In colorectal cancer cells, Cdk3 has been shown to promote epithelial-mesenchymal transition (EMT) via enhancing activity of AP-1 [185]. Another study in esophageal squamous cell carcinoma cells has shown that the oncogenic circular RNA circRNA_141539 exerts its function through sponging miR-4469 and enhancing activity of CDK3 [186]. Table 7 shows the function of CDK3 based on cell line studies.

**Table 7:** Function of CDK3 based on cell line studies

Tumor type	Targets/ Regulators and Signaling Pathways	Cell line	Function	References
Breast cancer	miR-4469/CDK3 axis and Wnt/β-catenin pathway	HEK293T, MCF7, T47D, MDA-MB-231 and BT549	↑↑ CDK3: ↓ metastasis, migration and invasion via inhibiting Wnt/β-catenin pathway CDK3 is a target of miR-4469	[181]
	miR-873/CDK3 axis	MCF-7, ZR75-1, T47D and MCF-7/TamR	NCTD treatment: ↑ sensitivity to tamoxifen, ↓ proliferation and tumor growth via miR-873/CDK3 axis NCTD was found to regulate ERα signaling by miR-873/CDK3	[182]
	HuR, CDK3	MDA-MB-231 and MCF-7	∆ HuR: ↓ CDK3 expression, ↓ proliferation, chemoresistance and ↑ apoptosis HuR increased proliferation and survival through stabilizing CDK3 transcripts	[187]
	miR-125a-3p/CDK3	MCF-7, MDA-MB-435 and MDA-MB-23	↑↑ miR-125a-3p: ↓ transcriptional activity of ERα, ↓ proliferation of ER + cells, and ↑ apoptosis and G1/S cell-cycle arrest via targeting CDK3	[183]
	miR-873/CDK3 axis	CF-7, ZR75-1, T47D, SKBR3, MDA- MB-231 and HEK293T	↑↑ miR-873: ↓ proliferation and ER activity via targeting CDK3	[184]
	CYP4Z1- and CYP4Z2P-3'UTRs, CDK3	MCF-7	↑↑ CYP4Z1- and CYP4Z2P-3'UTRs: ↓ tamoxifen resistance via targeting CDK3	[188]
Colorectal cancer	Cdk3/c-Jun	HEK293, HT29, SW620, HCT116, SW480 and HCoEpiC	↑↑ CDK3: ↑ metastasis, motility and invasion via EMT process Cdk3-phosphorylated c-Jun increased AP-1 activity	[185]
Esophageal squamous cell carcinoma	circRNA_141539/miR-4469/CDK3 axis	Kyse410, Kyse510, EC9706, ECA109 TE7 and Het-1A	↑↑ circRNA_141539: ↑ proliferation and invasion via regulating miR-4469/CDK3 axis	[186]
Hepatocellular carcinoma	miR-214, E2F2, CDK3 and CDK6	THLE3, QGY-7701, QGY-7703, HCC-9810, SMMC-7721, Hep3B, PLC/PRF5, Hep3B, QGY-7703, Bel-7402, Bel-7404, MHCC97L, MHCC97H, HCCLM3 and HCCLM6	↑↑ miR-214: ↓ proliferation and G1-S cell cycle arrest via targeting E2F2, CDK3 and CDK6	[189]
Leukemia	CDK3	HL-60, NB4, K562 and KG1	Benfotiamine:: ↓ proliferation and G1 cell cycle arrest via targeting CDK3	[190]
Lung cancer	HuR and miR-873/CDK3 and miR-125a-3p/CDK3 axis	A549 cells	↑↑ HuR: ↑ CDK3 levels, via increasing CDK3 mRNA stability and expression, thus increased stemness CDK3 was found to be a target of miR-873 and miR-125a-3p	[191]
Nasopharyngeal carcinoma	CDK3	5-8F, CNE1, CNE2, and NP-69	CDK3 was increased in CNE1, CNE2 and 5–8F NPC cell lines	[192]
Skin cancer	CDK3 and NFAT3	HEK293, T98G, HaCaT, A431, A375, G361, SK-MEL-5, and SK-MEL-28	CDK3 phosphorylated NFAT3 at serine 259 by interacting with NFAT3, thus increased the transactivation and transcriptional activity of NFAT3 CDK3-mediated phosphorylation of NFAT3 showed a significant role in skin cancer	[193]

*EMT* epithelial-mesenchymal transition, *NCTD* Norcantharidin, ∆ knock-down or deletion

### Animal studies

While a single study in breast cancer models has shown that up-regulation of CDK3 decreases metastatic abilities of breast cancer cells [181], other studies have shown that up-regulation of CDK3-targeting miRNAs miR-125a-3p [183] and miR-873 [184] leads to reduction of tumor growth. In xenograft models of colorectal cancer, up-regulation of CDK3 has been accompanied by enhancement of metastatic ability of cancer cells [185]. Table 8 summarizes function of CDK3 in animal models of cancer.

**Table 8:** Function of CDK3 in animal models of cancer

Tumor Type	Animal models	Results	References
Breast cancer	5 – 7-week-old female BALB/c nude mice	↑↑ CDK3: ↓ metastasis	[181]
	6-week-old female nude mice	NCTD treatment: ↓ tumor growth via miR-873/CDK3 axis	[182]
	4-week-old female BALB/c nude mice	↑↑ miR-125a-3p: ↓ tumor growth	[183]
	6- week-old female nude mice	↑↑ miR-873: ↓ tumor growth via targeting CDK3	[184]
Colorectal cancer	5– 6-week-old female nude BABL/c mice	↑↑ CDK3: ↑ metastasis	[185]
Hepatocellular carcinoma	4–5-week-old Male BALB/c-nu mice	↑↑ miR-214: ↓ tumor growth via targeting E2F2, CDK3 and CDK6	[189]
Skin cancer	6-week-old male BALB/c nu/nu mice	↑↑ NFAT3: ↑ tumor growth	[193]

*NCTD* Norcantharidin

### Investigations in clinical samples

Expression assays in breast cancer samples have shown that up-regulation of CDK3 is associated with chemoresistance [187]. In colorectal cancer samples, up-regulation of this member of CDK family has been associated with shorter progression-free survival and advanced TMN stage [186]. In clinical samples of nasopharyngeal carcinoma, up-regulation of CDK3 has been associated with tumor infiltration, lymph node metastasis and TNM staging [192]. Table 9 summarizes results of studies that reported association between up-regulation of CDK3 and clinical parameters.

**Table 9:** Dysregulation of CDK3 in clinical samples

Tumor type	Samples	Expression (Tumor vs. Normal)	Kaplan–Meier analysis (impact of regulators dysregulation)	Multivariate Cox regression analysis	Association of dysregulation of regulators with clinicopathologic characteristics	References
Breast cancer	37 cases of lymph node metastatic BC tissues, and 28 cases of lymph node non-metastatic BC tissues 194 cases of BC tissues and 59 cases of normal tissues	Up-regulation of CDK3 in primary tumor tissues	_	_	_	[181]
	30 PTANCT	Up-regulation of HuR and CDK3	_	_	chemoresistance	[187]
	37 PTANCT	Up-regulation of CDK3 and down of miR-125a-3p	_	_	_	[183]
Colorectal cancer	87 cases of PCC, 49 cases of MCC, and 52 cases of normal colon tissues	Up-regulation of CDK3 in MCC than PCC and in PCC than normal	_	_	TNM grade	[185]
	50 PTANCT	Up-regulation of circRNA_141539 (which regulated CDK3)	Shorter PFS	High levels of circRNA_141539 and low differentiation and stage III were found to be poor survival prognostic factors	TNM stage, T stage, and N stage, and negatively with histological grade	[186]
Hepatocellular carcinoma	GEO database (GSE22058: 96 PTANCT) 8 PTANCT	Down-regulation of miR-214/199a/199a* (which regulated CDK3)	Shorter OS	miR-214 expression was found to be an independent prognostic factor	_	[189]
Lung cancer	31 PTANCT	Up-regulation of HuR (which regulated CDK3)	_	_	_	[191]
Nasopharyngeal carcinoma	94 NPC tissues and 40 inflamed nasopharyngeal tissues	Up-regulation of CDK3 in NPC	_	_	infiltration, lymph node metastasis, tumor node metastasis, and TNM clinical staging	[192]
Skin cancer	65 tumor tissues and 9 normal tissues	Up-regulation of NFAT3	_	_	CDK3 levels were positively associated with both NFAT3 and phosphorylated NFAT3-Ser259	[193]

*PCC* primary colon cancer, *MCC* metastatic colon cancer, *TNM* tumor node metastasis, *PTANC* pairs of tumor samples and adjacent non-cancerous samples, *NPC* Nasopharyngeal carcinoma, *PFS* progression-free survival

## Cyclin-dependent kinase 4/6 (CDK4/6)

### Cell line studies

An in vitro study in AML has verified that suppression of CDK4/6 and autophagy enhances apoptosis in t(8; 21) AML cells in a synergic manner [194]. Similarly, CDK4/6 inhibition is a novel therapeutic modality for bladder cancer irrespective of RB1 status [195]. This treatment has reduced FOXM1 phosphorylation and exhibited synergy with cisplatin [195]. Another in vitro study in breast cancer cells has reported loss of the FAT1 as a mechanism for induction of resistance to CDK4/6 inhibitors. Mechanistically, FAT1 silencing has led to suppression of Hippo pathway in ER + cancer cells [196]. Single-cell assessment of CDK2 activity has confirmed difference in cell-cycle regulation between the luminal androgen receptor (LAR) subtype of triple negative breast cancer (TNBC) and basal-like cells. In fact, palbociclib-sensitive LAR cells leave mitotic cycle with low level of CDK2 activity, and enter a quiescent phase that needs activity of CDK4/6 for going back into cell-cycle. On the other hand, palbociclib-resistant basal-like cells leave mitosis and directly enter into a proliferative phase characterized by high level of CDK2 activity, circumventing the constraint point and the need for CDK4/6 activity. CDK4/6 inhibition has synergism with PI3 kinase inhibition in reduction of proliferation of PIK3CA-mutant TNBC cells, indicating that other subtypes of TNBC can be responsive to CDK4/6 inhibitors [197]. In breast and other solid tumors, CDK4/6 inhibitors could trigger anti-tumour immune responses [198]. Moreover, experiments in cervical cancer cells have shown that cyclin D-CDK4/6 inhibition enhances sensitivity of immune-refractory cancers through hindering the SCP3–NANOG axis [199]. Table 10 summarizes function of CDK4/6 based on cell line studies.

**Table 10:** Function of CDK4/6 based on cell line studies

Tumor type	Targets/ Regulators and Signaling Pathways	Cell line	Function	References
Acute B lymphocytic leukemia	miR-142-3p/ HOXA5 axis, CyclinD1, CDK4, Bax and Caspase-3	Hmy2-cir, Nalm6 and HOXA5	↑↑ miR-142-3p: ↓ proliferation and ↑ G1 phase arrest via targeting HOXA5 and reducing CyclinD1 and CDK4 and promoting the expression of Bax and Caspase-3	[200]
Acute myeloid leukemia	CDK4/6, MAP-ERK and PI3K-AKT-mTOR signaling pathway, LC3B-I to LC3B-II	Kasumi-1, SKNO-1, ML-2, HL-60, HEL, MV4-11, NB-4, KG-1a, Kasumi-6, KG-1, KO52, MOLM-16, U937, Kasumi-3, UF-1, CMK-86, MOLM-13, THP-1 and NOMO-1	Combination of CDK4/6 and autophagy inhibition: ↑ apoptosis in t(8;21) AML cells CDK4/6 inhibition: ↑ autophagy in t(8;21) AML cells	[194]
	miR-335-3p/EIF3E axis and CDK4, Cyclin D1, Bcl-2, p21 and Bad	THP-1 and U937	↑↑ miR-335-3p: ↓ proliferation and ↑ cell cycle G0/G1 arrest and apoptosis via targeting EIF3E and reducing the Cyclin D1, CDK4, c-Myc expression and elevating P21 and Bad expression	[201]
	miR-362-5p/ GAS7 axis and PCNA, CDK4, cyclin D1, and p21	TF-1, HL-60 and THP-1, HS-5	↑↑ miR-362-5p: ↑ proliferation via targeting GAS7 and increasing levels of PCNA, CDK4 and cyclin D1, but downregulating p21 expression	[202]
Bladder cancer	miR-124/CDK4 axis	HT1197, HT1376, J82, and 5637	↑↑ miR-124: ↓ growth and ↑ cell cycle arrest via targeting CDK4	[203]
	miR-195/CDK4 axis	SV-HUC-1, 5637 and BIU-87	↑↑ miR-195: ↓ cell migration, invasion, cloning efficiency, and EMT process via targeting CDK4	[204]
	miR-124/ CDK4 axis and E2F3, CDK4, Ki-67 and VEGF	Hek 293, SV-HUC-1, T24, 5637, J82 and UM-UC-3	↑↑ miR-124: ↓ cell viability, angiogenesis rate, proliferation, expression of E2F3, CDK4, Ki-67 and VEGF via targeting CDK4 and E2F3 ↑↑ CDK4: ↓ miR-124 inhibition of cell viability, angiogenesis, and cell cycle	[205]
	miR-1180-5p, p21, CDK4, CDK6, Cyclin D1 and Cyclin A2	Bladder cancer cell lines	↑↑ miR-1180-5p: ↓ proliferation via upregulating p21 and downregulating CDK4, CDK6, Cyclin D1 and Cyclin A2	[206]
	CDK4/6 and FOXM1	RT112, J82, 253J, 5637, UM-UC-1 and RT4	CDK4/6 inhibition: ↓ FOXM1 phosphorylation CDK4/6 inhibition showed synergy with CDDP	[195]
Breast cancer	CDK4/6, Hippo Pathway	MCF7, CAMA-1, HEK 293T, MCF7, T47D, and ZR-75–1	∆ FAT1: ↑ resistance to CDK4/6 inhibitors via the Hippo Pathway	[196]
	CDK4/6, PI3Kα and PTEN	T47D and MCF7	∆ PTEN: ↑ cross-resistance to CDK4/6 and PI3Kα inhibitors via increased AKT activation	[172]
	CDK4/6, AKT, cyclin D/CDK4-6/Rb and PI3K/AKT-mTOR pathways	MCF-7 and T47D, ZR-75-1, 182R-1, MPF-R,	Fulvestrant, CDK4/6i and AKTi triple combination: ↓ growth of breast cancer cells ∆ CDK4/6 and AKT: ↓ cyclin D/CDK4-6/Rb and PI3K/AKT-mTOR pathways	[207]
	PI3Kα and CDK4/6, PD-1 and CTLA-4	HCC70, HCC1806, MDA-MB-468 and AT3OVA	Combination of PI3Kα and CDK4/6 inhibitors: ↑ apoptosis, cell-cycle arrest, and tumor immunogenicity	[208]
	RB, Cyclin E, CDK2 and CDK4/6	MCF7 and T47D	Low levels of RB and high levels of Cyclin E were observed in CDK4/6 inhibitor-resistant cells	[98]
	Wnt signaling pathway, MYC, and β-catenin	MDA-MB-231, CAL-148, MDA-MB-453, MDA-MB-157, MDA-MB-436, HCC1937, SUM149, MDA-MB-468, HEK293T	PARPi olaparib and the CDK4/6i palbociclib: ↓ HR during the G2 phase, ↓ tumour growth, ↓ MYC expression through the Wnt pathway, and ↑ DNA damage	[209]
	CDK4/6-USP51-ZEB1 axis	MDA-MB-231, 293T, and SUM-159	∆ CDK4/6: ↓ tumor metastasis by destabilizing the ZEB1 protein CDK4/6 stabilizes ZEB1 by phosphorylation and activation of USP51	[210]
	CDK4/6, CCND1	MCF-7, ZR-75-1, and HCC-1428	Combination of ZEN-3694 with CDK4/6 inhibition: ↓ proliferation and ↑ apoptosis	[211]
	CDK4/6; HLA	MDA-MB-231 and MCF7, CAL-51, SK-BR-3, HCC1143, BT-474, MDA-MB-453, BT-20, T-47D, HCC1143, BT-549, Hs587T, HEK293, HEK293T, HFF-1, MCF 10 A, WI-38, IMR-90, and HeLa	CK1ε inhibition not only inhibits RB1 from degradation, but also inhibits CDK4/6i-induced CDK6 up-regulation via modulating SP1 protein stability, so increasing CDK4/6i efficacy	[212]
	CDK4/6, Cyclin D1, HLA ligands (PSMC1)	MCF7 and T47D	Low-dose of CDK4/6 inhibitor: ↑ HLA class I surface expression in breast cancer cells HLA ligands induced by CDK4/6i were found to be derived from proteins enriched in G1/S cell cycle transition	[213]
	PI3K/mTOR signaling, CDK4/6-p-Rb signaling pathway	MCF7 and HCC1500, EFM19	Acquired resistance to CDK4/6 inhibitor monotherapy was found to be correlated with loss of dependence on pRb and induction of PI3K/mTOR signaling Targeting PI3K/mTOR signaling dominates resistance to CDK4/6 inhibitors	[214]
Breast cancer	CDK4/6, HMGB1, TLR4 and NF-κB pathway	MCF‐7 and T47D	↑↑ HMGB1: ↑ tamoxifen resistance by combining with the TLR4 and NF-κB pathway CDK4/6 inhibition: ↓ expression of HMGB1 and ↓ TLR4-NF-κB pathway, and in turn ↓ tamoxifen resistance	[215]
	Cdk4/6 and TSC2 and mTORC1	MCF7	Cdk4/6 inhibition: ↓ proliferation partly via TSC2 and mTORC1 Cdk4/6 Regulates mTORC1 via the TSC Cdk4/6 was found to phosphorylate TSC2, and in turn regulate mTORC1 via the TSC	[216]
	CDK4/6 and PARP	MDA-MB-231 and SUM-159	CDK4/6 and PARP dual inhibitor, ZC-22: ↑ cell cycle arrest and ↑ DNA damage ZC-22 was more effective than the combination of PARPi Olaparib and CDK4/6i Abemaciclib	[217]
	CDK4/6, p21	MDA‐MB‐231 and MCF‐7	Abemaciclib and ABT-263 combination: ↓ viability of MDA-MB-231 cells, but not MCF-7 cell, and ↓ cytoplasmic p21 expression in MDA‐MB‐231 cells, ↑ caspase-dependent apoptosis in MDA-MB-231 cells ∆ p21: ↑ sensitivity of MCF‐7 cells to TRAIL	[218]
	CDK4/6, CDK2, RB1	12 RB1 wild-type TNBC cell lines and one RB1 mutant cell line (BT549), MFM223 cell, MFM223pR cells, MES CAL51	LAR subtype of TNBC was found to be sensitive to CDK4/6 inhibitors Cell lines with palbociclib sensitivity showed low post-mitotic CDK2 activity The proliferative CDK2 high subpopulation had resistance to CDK4/6 inhibitors	[197]
Breast cancer	miR-124/CDK4 axis	MCF-7, Bcap-37, and MDA- MB-435S	↑↑ miR-124: ↓ cell viability, proliferation, and cell cycle progression via targeting CDK4	[219]
	miR-623, XRCC5, CDK4/6 and PI3K/AKT and Wnt/β-catenin signaling pathways	MDA-MB-453 and MCF7	↑↑ miR-449a/b: ↓ proliferation, migration, invasion and ↑ apoptosis via targeting XRCC5 and reducing CDK4/6 MiR-623 suppressed the activations of PI3K/AKT and Wnt/β-catenin signaling pathways induced by XRCC5	[220]
	AFAP1-AS1/ miR-545/CDK4 axis	MDA-MB-231 and BT-549	AFAP1-AS1is involved in TNBC pathogenesis via regulating miR-545/CDK4 axis	[221]
	MALAT1-miR-124-CDK4/E2F1 signaling pathway and CDK4	MCF-7, MDA-MB-435S, MDA-MB-231, ZR-75-1, HSS578T, HCC1937 and BCAP-37, and MCF-10A	↑↑ miR-124: ↓ proliferation and ↑ cell cycle G0/G1 phase arrest via targeting CDK4/E2F1 signaling pathway MALAT1 was found to inhibit miR-124 and increase the expression of CDK4	[222]
	miR-519d-3p	MDA-MB-231 and HCC1937	↑↑ miR-519d-3p: ↓ proliferation, colony formation, migration, invasion and ↑ G0/G1 phase via targeting LIMK1 and reducing expression of CDK4, 6/Cyclin D1, and CDK2/Cyclin E1	[223]
	miR-1301-3p/ICT1 axis and CDK4, Cyclin D1, Bcl-2, p21, Bad and Bax	MCF-7, T-47D, MDA-MB-231, MDA-MB-468, and MCF-10A	↑↑ miR-1301-3p: ↓ proliferation, growth and ↑ G0/G1 phase arrest and apoptosis via targeting ICT1, and reducing the expression of CDK4, Cyclin D1, Bcl-2, but elevating p21, Bad and Bax levels	[224]
	miR-200b-3p and miR-429-5p, cyclin D1/CDK4/CDK6 and cyclin E1/CDK2, and LIMK1/CFL1 pathway	MDA-MB-231, HCC1937, MCF-7 and MCF-10	↑↑ miR-200b-3p and miR-429-5p: ↑ G2/M and G0/G1 cell cycle arrest via downregulating cyclin D1/CDK4/CDK6 and cyclin E1/CDK2, and ↓ proliferation, migration, and invasion via the LIMK1/CFL1 pathway	[225]
	miR-34c, CCND1, CDK4 and CDK6	MDA-MB-231, MDA-MB-468, BT-549 and T47D	↑↑ miR-34c: ↓ proliferation and ↑ cell death and G2/M phase arrest via downregulating miR-34 targets CCND1, CDK4 and CDK6	[226]
Breast cancer and other solid tumors	CDK4/6	BT474, SKBR3, MDA-MB-361, MDA-MB-453, and MCF7, MMTV-PyMT-S2WTP3, B16-OVA, and CT-26	∆ CDK4/6: ↓ proliferation, ↑ anti-tumour immunity and cell cycle arrest	[198]
Cervical cancer	SCP3, AKT/cyclin D1–CDK4/6 signaling, NANOG and cyclin D1–CDK4/6/E2F1 axis	CaSki	SCP3 induces immune-resistant and stem-like features through AKT/cyclin D1–CDK4/6 signaling SCP3 enhanced transcription of NANOG through the cyclin D1–CDK4/6/E2F1 axis	[199]
	circ_0000326/miR-338-3p/CDK4 axis	Hela, Caski, SiHa, SW756 and C-33A	∆ circ_0000326: ↓ proliferation, migration and cell cycle progression via miR-338-3p/CDK4 axis	[227]
Clear cell renal cell carcinoma	miR-1, CDK4, CDK6, Caprin1 and Slug	ACHN, 786-O, SN12-PM6 and HK-2	↑↑ miR-1: ↓ proliferation, motility, migration and invasion via targeting CDK4, CDK6, Caprin1 and metastasis related gene Slug	[228]
	DMDRMR, IGF2BP3, CDK4	786-O, 769-P, ACHN, and Caki-1, HK2, and HEK293T	DMDRMR enhanced the G1-S transition, and promotes cell proliferation via cooperating with IGF2BP3 to regulate target genes including CDK4 in an m6A-dependent manner	[229]
	miR-206/ CDK4, CDK9 and CCND1 axis	ACHN, 786-O, SN12PM6 and HK-2	↑↑ miR-206: ↓ proliferation and ↑ cell cycle arrest via directly targeting cell cycle related gene CDK4, CDK9 and CCND1	[230]
Colorectal cancer	HAGLR/miR-185-5p/CDK4 and CDK6 axis	FHC, DLD-1, SW620 HCT-116, LOVO, and SW480	∆ HAGLR: ↓ proliferation, and ↑ apoptosis via regulating miR-185-5p/CDK4 and CDK6 axis	[231]
	miRNA-20b-5p/ CCND1/CDK4/FOXM1 axis	HCT-116, SW480, and HT29, 293T cells, and 3T3	↑↑ miRNA-20b-5p: ↓ cell cycle, migration, and invasion in but had no effect on apoptosis via targeting CCND1 and regulating CCND1/CDK4/FOXM1 axis	[232]
	MCM3AP-AS1/ miR-545/CDK4 axis	CR4	↑↑ MCM3AP-AS1: ↑ cell cycle progression and proliferation, ↓ G1 arrest via regulating miR-545/CDK4 axis	[233]
	miR-142-3p/CDK4 axis	HEK293T, HT29 and SW116	↑↑ miR-142-3p: ↓ viability and colony formation and ↑ cell cycle arrest via targeting CDK4	[234]
	miR-6883-5p and miR-149*, CDK4/6 and CDK4/6-FOXM1 signaling	HCT116, RKO, HT-29, and SW480	↑↑ miR-6883-5p and miR-149*: ↓ cell growth, ↑ G0-G1 phase cell-cycle arrest and ↑ apoptosis by partially targeting CDK4/6 MiR-6883-5p and miR-149* combinations: ↓ CDK4/6-FOXM1 signaling	[235]
	miR-875-5p/ EGFR axis, cyclin D1, cyclin D2, CDK4, p57and p21	DLD1, HCT116, LOVO, RKO, LS174T, HCT8, HR28348, HT29, SW620, SW480 and NCM460	↑↑ miR-875-5p: ↓ cell proliferation, migration, invasion, and ↑ apoptosis via targeting EGFR and downregulating cyclin D1, cyclin D2, CDK4, Bcl2 and upregulating protein cleaved caspase-3, p57and p21	[236]
	uc.77-/ miR-4676-5p/FBXW8/CDK4 axis	HCT116, HT-29, LoVo, and SW620	↑↑ uc.77-: ↓ proliferation and ↑ G0/G1 phase arrest via targeting miR-4676-5p and upregulating FBXW8, in turn FBXW8-mediated CDK4 Protein degradation	[237]
	LINC00665, miR-126-5p, and cyclin D1, CDK4, Rb	DLD1, RKO, HCT116, LOVO, SW480 and NCM460	∆ LINC00665: ↓ proliferation and ↑ apoptosis via upregulating miR-126-5p, thus reducing cyclin D1, CDK4, Rb	[238]
	miR-29a-3p/RPS15A axis and CDK4, Cyclin D1, p21, Bax and Bcl-2	DLD-1, RKO, SW480, and HCT116, and FHC	↑↑ miR-29a-3p: ↓ proliferation, ↑ cell cycle arrest and apoptosis via targeting RPS15A and regulating CDK4, Cyclin D1, p21, Bax and Bcl-2	[239]
Epithelial ovarian cancer	PCAT-1, cyclin D1 and CDK4	SKOV-3, OVCAR-3, HEY-A8, and HO8910-PM	∆ PCAT-1: ↓ proliferation, migration and invasion, but ↑ G0/G1 phase arrest via decreasing levels of cyclin D1 and CDK4	[240]
Esophageal cancer	miR-486/ CDK4/BCAS2 axis	KYSE150, EC9706 and TE-9, and Het-1A	↑↑ miR-486: ↓ colony formation, migration and invasion, ↑ G0/G1 phase arrest and apoptosis via targeting CDK4/BCAS2	[241]
	miR-124/CDK4 axis	TE-1	↑↑ miR-124: ↓ tumor growth and ↑ apoptosis	[242]
Esophageal squamous cell carcinoma	miR-1/MET/cyclin D1/CDK4 axis	Het-1A, QBC939, HepG2, and 293T	↑↑ miR-1: ↓ proliferation, and ↑ apoptosis via targeting MET, cyclin D1, and CDK4	[243]
Ewing's sarcoma	CDK4/6, IGF1R and PI3K/mTOR signaling	A673, SKNEP1, SKNMC, CADOES1, TC32, SKPNDW, AEW541, and GDC0941	Combination of CDK4/6 and IGF1R inhibition: ↓ cell cycle progression and PI3K/mTOR signaling	[173]
	DLX6-AS1/miR-124-3p/CDK4 axis	SK-ES-1, A673, RD-ES, and MSCs	∆ DLX6-AS1: ↓ proliferation, and ↑ apoptosis via regulating miR-124-3p/CDK4 axis	[244]
Gastric cancer	CDK4/6, PAK1, PDK1-AKT pathway,	SGC-7901 and MKN-45	CDK4/6 inhibition: ↓ cell viability and ↓ PAK1 expression ∆ PAK1: ↑ cell sensitivity exposed to CDK4/6 inhibitor and ↑ DNA damage ↑↑ PDK1: ↓ effect of PAK1 deletion on DNA damage ↓ sensitivity towards CDK4/6 inhibitor and ↓ cell cycle arrest caused by PAK1 depletion	[245]
	miR-449a/b/CDK4/6, E2F1, and CDKs-pRb-E2F1 signaling pathway	BGC-823 and GES-1	↑↑ miR-449a/b: ↓ proliferation and migration and ↑ apoptosis via targeting CDK4 and CDK6	[246]
	miR-1301-3p, SIRT1, Cyclin D1, CDK4, c-Myc, P21	GES-1, HEK-293T, SGC-7901 and MGC-803, CCK-8	↑↑ miR-1301-3p: ↑ proliferation and cell cycle progression via targeting SIRT1 and elevating the Cyclin D1, CDK4, c-Myc expression and reducing P21 expression	[247]
	miR-486-5p, SMAD2, CDK4, and ACTR3	GC9811, GC9811-P, HMrSV5	↑↑ miR-486-5p: ↓ EMT process via reducing SMAD2, CDK4, and ACTR3	[248]
	miR-34a, Bcl-2, CDK4, and cyclin D1	SGC-7901 cells	Curcumin: markedly ↑↑ miR-34a, ↓ proliferation, migration, and invasion, cell cycle progression in G0/G1-S phase and via downregulating the Bcl-2, CDK4, and cyclin D1 protein expression	[249]
	miR-143/ DNMT3A axis and Cyclin D1, CDK4 and CDK6	MKN28, MKN-45, BGC-823, SGC-7901 and MGC803 and GES-1	↑↑ miR-143: ↓ proliferation, invasion, and cell cycle progression via targeting DNMT3A and reducing Cyclin D1, CDK4 and CDK6	[250]
	RASSF1A/miR-711/CDK4 axis	SGC-7901	↑↑ RASSF1A: ↓ proliferation, viability, migration, invasion and ↑ G1 phase arrest via upregulating miR-711 and in turn downregulating CDK4	[251]
	Linc-ROR/miR-212-3p/FGF7 axis and CDK4, CDK6, Cyclin D1, N-Cadherin, Vimentin, MMP-9, MMP-2, P21, P27, E-Cadherin, and CK-19	AGS and MGC-803	∆ Linc-ROR: ↓ proliferation, migration, and invasion via miR-212-3p/FGF7 axis and downregulating CDK4, CDK6, Cyclin D1, N-Cadherin, Vimentin, MMP-9, MMP-2, but upregulating of P21, P27, E-Cadherin, CK-19	[252]
Gastric cancer	miR-29a-3p, CDK2, CDK4, and CDK6	GES-1, SGC-7901, AGS, MCG803, and BGC-823	↑↑ miR-29a-3p: ↓ proliferation via downregulating the expression of CDK2, CDK4, and CDK6	[253]
	GCRL1/miR-885-3p/CDK4 axis	SGC-7901, GES-1, MGC-803, BGC-823, and AGS	↑↑ GCRL1: ↑ proliferation, migration and invasion by targeting miR-885-3p, and positively regulating CDK4	[254]
Glioblastoma	CDK4/6, Rb1, and ↓ miR-17˜92 family, E2F cell cycle pathway	GSC lines	Palbociclib, CDK4/6 inhibitor: ↓ Rb1 phosphorylation and ↓ miR-17˜92 family and paralog expression in the sensitive PN GSC lines, and ↑ proneural-mesenchymal transition	[255]
	CDK4/6, c-Met/TrkA-B pathways	G88 cells and GBM cells	Combination of CDK4/6 inhibitor, abemaciclib, with c-Met/Trk inhibitor, altiratinib: ↑ cell cycle arrest and ↑ cytotoxicity via enhanced apoptosis	[256]
	miR-129/CDK4/6 and MDM2 axis	U87MG, 251, U87, and HEK293	↑↑ miR-129: ↓ cell cycle and growth via targeting CDK4/6 and MDM2 axis	[257]
Glioblastoma multiforme	miR-124-CDK4 axis	SWO-38 and U251	∆ CDK4: ↑ radiosensitivity ↑↑ miR-124: ↑ radiosensitivity via targeting CDK4	[258]
	miR-138, EZH2, CDK6, E2F2, E2F3, and EZH2-CDK4/6-pRb-E2F1 pathway	NHA, 87MG, U251MG, A172, T98G, U118 and SHG-44	↑↑ miR-138: ↓ proliferation but ↑ G1/S cell cycle arrest via directly targeting EZH2, CDK6, E2F2 and E2F3, and in turn blocked EZH2-CDK4/6-pRb-E2F1 loop	[259]
	circMMP9/ miR-124/CDK4 and AURKA axis and eIF4A3	U251, SHG44, A172, SNB19 and U87	∆ circMMP9: ↓ proliferation, migration, and invasion vi regulating miR-124/CDK4 and AURKA axis eIF4A3 was found to promote circMMP9 expression	[260]
Glioma	CDK4/6 and RB	U87, U251, H4, A172, and NHAs	∆ CDK4: ↓ colony formation and proliferation, and ↑ apoptosis and sensitivity to TMZ RB phosphorylation mediated by CDK4 showed oncogenic function in glioma Selective inhibitors of CDK4/6: ↓ proliferation and ↑ apoptosis	[261]
	HMMR-AS1/ miR-7/CDK4 axis	LN229, T98 and A172	∆ HMMR-AS1: ↓ cell viability, invasion, and colony formation via upregulating miR-7 and reducing CDK4 Sevoflurane treatment: ↓ glioma cell progression via reducing HMMR-AS1 and increasing miR-7, thus downregulating CDK4 ↑↑ miR-7: ↓ cell viability, invasion, and colony formation ability via reducing CDK4	[262]
H. pylori related gastric cancer	miR-101/ SOCS2 axis and c-myc, CDK2, CDK4, CDK6, CCND2, CCND3, and CCNE2, p14, p16, p21 and p27	GES-1, MKN45 and 7901	↑↑ miR-101: ↓ proliferation and colony formation and ↑ G1-phase arrest via targeting SOCS2 and downregulating c-myc, CDK2, CDK4, CDK6, CCND2, CCND3, and CCNE2	[263]
Head and neck mucosal melanoma	CDK4	ME OMM cell line	CDK4 knockdown in ME cells led to delayed G1/S cell cycle phase transition Abemaciclib and dacarbazine synergistically inhibited ME cells	[264]
Head and neck squamous cell carcinoma	CDK4/6, mTOR and stat3 pathways, IL6-stat3 axis	Cal27, HSC3 and HSC6	Combination of CDK4/6 inhibitor, LY2835219, and metformin: ↑ cell cycle arrest and ↓ colony formation, viability, growth SASP which is induced by LY2835219 could upregulate cancer stemness, but it can be attenuated in combination with metformin	[265]
Hepatocellular carcinoma	CDK4/6 and PI3K/AKT signaling pathway	Huh7, HepG2 and Hep3B	Aminoquinol, a new CDK4/6 and PI3K/AKT inhibitor: ↓ viability, ↑ apoptosis, and ↑ G1 phase arrest	[174]
	CDK4/6-Rb-myc and mTORC1/p70S6K signaling	HepG2, HUH7, PLC/PRF-5, HEP3B	Combination of Palbociclib with Regorafenib: ↓ spheroid cell growth and ↓ cell migration/ and invasion, and ↑ cell death The combination teraphy was found to be more effective than single treatments also under hypoxia	[266]
	circ_0001588/miR-874/CDK4 axis	SK-Hep-1, Hep-3B, HepG2, BEL-7402, and MHCC-LM3, and LO2	∆ circ_0001588: ↓ proliferation, migration, and invasion vi regulating miR-874/CDK4	[267]
	hsa_circ_0016788/miR-486/CDK4 axis	HepG2, Hep3B, Huh7, HCCLM3, MHCC97L, LO2	∆ hsa_circ_0016788: ↓ proliferation, invasion and ↑ apoptosis via regulating miR-486/CDK4 axis	[268]
	miR-498/FOXO3 axis and Cyclin D, CDK4	HepG2 and Huh7	↑↑ miR-498: ↓ proliferation, migration, invasion, ↑ cell cycle arrest and apoptosis via inducing FOXO3 expression and regulating Cyclin D, CDK4	[269]
	CCDC144NL-AS1/ miR-940/WDR5 axis and MMP2, MMP9, CDK1, CDK2, and CDK4	Huh-7, HepG2, Hep3B, SMMC-7721, MHCC97H, SNU-368, HCCLM3, and L02	↑↑ CCDC144NL-AS1: ↑ proliferation, invasion and ↓ apoptosis via miR-940/WDR5 axis CCDC144NL-AS1 and WDR5 upregulated MMP2, MMP9, CDK1, CDK2, and CDK4 expression	[270]
	miR-34a, p-p53, SIRT1, cyclin D1, CDK4, CDK6, BCL-2, MDR1/P-gp and AXL proteins	HepG2	miR-34a combined with treatment with doxorubicin: ↓ proliferation, viability, ↑ G1 phase arrest and apoptosis via downregulating expression levels of p-p53, SIRT1, cyclin D1, CDK4, CDK6, BCL-2, MDR1/P-gp and AXL proteins	[271]
	miR-497, miR-195, CCNE1, CDC25A, CCND3, CDK4, and BTRC	Hep G2, Hep 3B, HLE, Huh7, JHH-4, and sK-Hep-1	↑↑ miR-497 and miR-195: ↓ cell growth and↑ G1 arrest CCNE1, CDC25A, CCND3, CDK4, and BTRC were found to be direct targets for miR-497 and miR-195	[272]
	circSP3/ miR-198/CDK4 axis	Hep-3B, Huh-7, Bel-7402, SMMC-7721 and HL-77O2	↑↑ circSP3: ↑ proliferation, migration and invasion via targeting miR-198 and inducing CDK4	[273]
	VPS9D1-AS1/HuR/CDK4 signaling axis	HepG2	∆ VPS9D1-AS1: ↓ proliferation and colony formation but ↑ apoptosis VPS9D1-AS1 was found to bind to the HuR protein and thus increase the stability and expression of the CDK4 mRNA	[24]
Kaposi’s sarcoma–associated herpesvirus	miR-34a-5p/ c-fos axis, CDK4/6, cyclin D1, MMP2, MMP9	SH-SY5Y and 293T	↑↑ miR-34a-5p: ↓ proliferation and migration, and ↑ G1 cell cycle arrest via targeting c-fos, thus down-regulating CDK4/6, cyclin D1, MMP2, MMP9	[274]
Leiomyosarcoma	CDK4/6, Rb	SK-LMS-1 and SK-UT-1	Palbociclib treatment: ↓ protein levels of Phospho-Rb, ↓ proliferation, and ↓ G0/G1-phase arrest with decreased S/G2 fractions in SK-LMS-1 but SK-UT-1 did not respond	[275]
Lung cancer	CDK4/6 and PAKs	H157, H322, H1299, H2170, A427, HCC4006, H1648, HCC827, H1437, H1944, H2172 and HBEC	CDK4/6 and PAKs inhibitor combination: ↑ apoptosis	[276]
	CDK4/6 and RB	H1975 and H1975OR	Combination of CDK4/6 inhibitor palbociclib and osimertinib: ↓ resistance of osimertinib	[277]
	LINC01194/ miR-486-5p/CDK4 axis	A549, H1299, H460, H1975, and BES-2B	∆ LINC01194: ↓ proliferation, migration and invasion via regulating miR-486-5p/CDK4 axis	[278]
	hsa_circ_0014235/miR-520a-5p/CDK4 axis	A549, H1299, and 16HBE	↑↑ hsa_circ_0014235: ↑ DDP chemoresistance, proliferation, migration and invasion via regulating miR-520a-5p/CDK4 axis	[279]
	miR-613/CDK4 axis	HEK293T, A549 and SPCA1	↑↑ miR-613: ↓ cell viability and colony formation and cell cycle arrest via targeting CDK4	[280]
	miR-34b-3p/CDK4 axis	A549, H1299, and BEAS‐2B	↑↑ miR-34b-3p: ↓ proliferation, ↑ cell cycle arrest and apoptosis via targeting CDK4	[281]
	circRNA_001010/miR-5112/ CDK4 axis	A549	↑↑ circRNA_001010: ↑ proliferation, migration and invasion and ↓ apoptosis via regulating miR-5112/ CDK4 axis	[282]
	miR-143, miR-506, CDK1, CDK4, and CDK6	H69-AR, Calu3, H358, and H1975	Combinatorial treatment with miR-143 and miR-506: ↓ CDK1, CDK4, and CDK6, cell cycle progression and ↑ apoptosis	[51]
	miR-340/ CDK4 axis	A549, H1299, H460, and 16HBE	↑↑ miR-340: ↓ proliferation via targeting CDK4	[283]
	miR-486-5p/CDK4 axis	BEAS-2B, A549, H1650, PC-9, 95-D and SPCA-1	∆ CDK4: ↓ proliferation, and ↑ apoptosis ↑↑ miR-486-5p: ↓ proliferation and cell cycle progression via targeting CDK4	[284]
	miR-326, CCND1, cyclin D1, cyclin D2, CDK4, p57and p21	A549, SPC-A-1, H1299, SK-MES-1, 95D, and HELF	↑↑ miR-326: ↓ cell proliferation, migration, invasion, and ↑ apoptosis via targeting CCND1 and downregulating expression levels of cyclin D1, cyclin D2, CDK4 and upregulating of p57 and p21	[285]
	miR-134/ CCND1 axis and cyclin D1, cyclin D2, CDK4, p57and p21	A549, SPC-A-1, H1299, SK-MES-1, NCI-H520, 95D, and HELF	↑↑ miR-134: ↓ cell growth, cell viability, colony formation, migration and invasion and ↑ apoptosis via targeting CCND1 and reducing cyclin D1, cyclin D2, CDK4 and up-regulation of p57and p21	[285]
	miR-98, TWIST- Akt-CDK4/CDK6 and TWIST-Akt-bcl2/Bax pathways	A549 and NCI-H23	↑↑ miR-98: ↓ proliferation, invasion via inhibiting TWIST- Akt-CDK4/CDK6 and ↑ apoptosis via activating TWIST-Akt-bcl2/Bax pathway	[286]
	miR-1290/ IRF2 axis and CDK2 and CDK4	A549, H1299, SPC-A1, H1970 and H460, and BEAS-2B	↑↑ miR-1290: ↑ proliferation, colony formation and invasion via targeting IRF2 and upregulating CDK2 and CDK4	[287]
	circHIPK3/miR-124 axis and SphK1, STAT3 and CDK4	A549 and BEAS-2B	↑↑ circHIPK3: ↑cell survival and proliferation via targeting miR-124 and upregulating SphK1, STAT3 and CDK4	[288]
	miR-593, SLUG/protein kinase B (Akt)/cyclin D1/CDK4 or CDK6 signaling pathway and SLUG/Akt/Bcl-2/BAX signaling pathway	A549, NCI-H1299, NCI-H358 and NCI-H1993	↑↑ miR-593: ↓ proliferation via inactivating the SLUG/protein kinase B (Akt)/cyclin D1/CDK4 or CDK6 signaling pathway	[289]
	SART3, miR-34a, and CDK4/6	A549, HEK293T cells, H1299 and NTERA-2	SART3 overexpression: ↑ miR-34a levels, ↓ the miR-34a target genes CDK4/6, thus caused G1 phase arrest	[290]
	LncSENCR/miR-1-3p/CDK4/6 axis	A549, SPC-A1, H1299, H1650, H1975 and PC-9, and 16HBE	∆ lncSENCR: ↓ proliferation via targeting miR-1-3p and upregulating CDK4/6	[291]
	miR-545, cyclin D1 and CDK4	A549, HFL1 and NCI-H460	↑↑ miR-545: ↓ proliferation but ↑ G0/G1 phase arrest and apoptosis via targeting cyclin D1 and CDK4	[292]
	linc00703, cyclinD1 and CDK4	A549, H226, PC-9, H358 and BEAS-2B	↑↑ linc00703: ↓ proliferation, colony formation, but ↑ G1/G0 phase arrest and apoptosis via reducing expressions of cyclinD1 and CDK4	[293]
Lung cancer	circ_0007766 and Cyclin D1/Cyclin E1/CDK4 pathway	SPCA-1	∆ circ_0007766: ↓ proliferation, migration, but ↑ G0/G1 phase arrest and apoptosis via reducing expression of Cyclin D1/Cyclin E1/CDK4	[294]
Medulloblastoma	CDK4/6, PI3K, and FGFR	DAOYand UW228-3,	PI3K, FGFR, and CDK4/6 inhibition: ↓ viability and proliferation PI3K, FGFR, and CDK4/6 inhibition and combination with irradiation could have positive effects	[295]
	HOTAIR/miR-483-3p/CDK4 axis	Daoy and D341	∆ HOTAIR: ↓ proliferation, and ↑ apoptosis via regulating miR-483-3p/CDK4 axis	[296]
	miR-221-3p/ EIF5A2 axis and CDK4, Cyclin D1, Bcl-2 and Bad	D341: No. HTB-185; D283 Med: No. HTB-187, and DAOY	↑↑ miR-221-3p: ↓ proliferation and ↑ G0/G1 arrest and apoptosis via targeting EIF5A2 and downregulating CDK4, Cyclin D1 and Bcl-2 and increasing Bad expression	[297]
Melanoma	CDK4/6, PRMT5-MDM4 axis	A375, HT144, CHL1, MCF7, MDA-MB-231, HS578T, and HEK293T, C002, D04, A11, and C067	∆ CDK4/6 and PRMT5: ↑ efficacy of palbociclib in both naive and resistant models and ↓ emergence of resistance	[298]
	CDK4/6 and p53 pathway	WM266.4 and A375 BRAF mutant melanoma cells	∆ CDK4/6: ↑ mitochondrial metabolism in BRAF V600 melanoma via a p53 dependent pathway	[299]
	MEK, CDK4/6, NRAS, BRAF	WM3629, WM3670, WM3060, WM1366, D04, Sk-Mel-2, MM485, MM415, MaMel27II, A375, A2058, Sk-Mel28, MM466, and MaMel30I	Combination of MEK/CDK4,6 inhibitors: ↓ cell viability in a number of NRAS mutant melanoma cells and ↓ tumor growth in BRAF mutant and ‘wild-type’ melanoma cell lines	[300]
	CDK4/6, VEGF-A	518A2 and LNM1	∆ CDK4 or CDK6: ↓ proliferation and migration, ↓ VEGF-A expression and ↓ stimulation of endothelial cell growth CDK4/6 inhibition: ↓ proliferation and ↓ angiogenesis	[301]
	CDK4/6, MEK	Mouse D4M3.A, Human SKMEL207	CDK4/6i alone and in combination with MEKi could enhance expression of CD137L, a T-cell costimulatory molecule on immune cells MEK inhibition: ↓ phospho-ERK1/2 CDK4/6 inhibition: ↓ phospho-RB1 amounts	[302]
Melanoma	CDK4/6, RTK-RAS-RAF and RTK-PI3K-AKT pathways and NRAS	Hs936T, Hs944T, MELJUSO, SKMEL30, IPC298, SKMEL-2	NRAS-mutant melanomas showed resistance to genetic ablation of NRAS or combination MEK1/2 and CDK4/6 inhibition	[303]
	hsa_circ_0025039/ miR-198/CDK4 axis	HEMn, A375, SK-MEL-1, A2058 and 293T cell	∆ hsa_circ_0025039: ↓ proliferation, colony formation, invasion and glucose metabolism via regulating miR-198/CDK4 axis	[304]
	miR-206, CDK4, Cyclin D	A375, MALME-3M, RPMI7951, SK- MEL-2, and SK-MEL-5	↑↑ miR-206: ↓ proliferation, migration, invasion, but ↑ G0/G1 phase arrest via targeting CDK4, Cyclin D	[305]
Multiple myeloma	Lnc-Pvt1/miR-486/ CDK4 and BCAS2 axis	CI-H929, U-266, LP-1 and RPMI-8226 and human normal plasma cells	∆ Lnc-Pvt1: ↓ proliferation, invasion and ↑ apoptosis via regulating miR-486/ CDK4 and BCAS2 axis	[306]
	miR-338-3p/CDK4 axis	NCI-H929, MM1S, U266, and RPMI-8266	↑↑ miR-338-3p: ↓ proliferation, cell cycle progression, but ↑ apoptosis via targeting CDK4	[307]
Myxoid liposarcoma	FUS-CHOP/miR-486/CDK4 axis	1955/91 cells	∆ FUS-CHOP: ↓ growth, and ↑ apoptosis via regulating miR-486/CDK4 axis	[308]
Nasopharyngeal carcinoma	CDK4/c-Myc/miR-16/CCND1 pathway	5-8F and HONE1	∆ CDK4: ↓ expression of c-Myc, whish suppresses the miR-16 expression ↑↑ miR-16: ↓ CDK4 expression by repressing CCND1	[309]
	miR-539/CDK4 axis	HEK293T, SUNE-1 and CNE-1	↑↑ miR-539: ↓ cell growth and ↑ cell cycle arrest via targeting CDK4	[310]
	RP11-624L4.1 and CDK4/6-Cyclin D1-Rb-E2F1 pathway	NP69, CNE1, CNE2, 6-10B, 5-8F, HNE3, and C666-1	↑↑ RP11-624L4.1: ↑ proliferation via the CDK4/6-Cyclin D1-Rb-E2F1 pathway	[61]
Oral squamous cell carcinoma	MMP1, miR-188-5p, and CDK4 SOX4 axis	Tca8113 and HEK-293T	↑↑ MMP1: ↑ growth, motility, migration and invasion via regulating miR-188-5p, and CDK4 SOX4 axis	[311]
	miR-198/CDK4 axis	Cal-27, SCC-9, SCC-25, and HaCaT	↑↑ miR-198: ↓ proliferation, invasion, EMT process, and ↑ apoptosis via targeting CDK4	[312]
	miR-519d-3p/ CCND1 axis, CDK4, CDK6	CAL-27 and HN-6	↑↑ miR-519d-3p: ↓ cell viability and proliferation, ↑ G0/G1 phase arrest via targeting CCND1 and downregulating the expressions of CDK4, CDK6	[313]
	miR-9 and CDK 4/6 pathway	Tca8113	↑↑ miR-9: ↓ cell growth, migration and colony formation, and ↑ cell arrest and apoptosis via CDK 4/6 pathway CDK6 was found to be a target of miR-9	[314]
Osteosarcoma	miR-590-3p/ CDK4 axis	SaOS2, U2OS, MG63 and HOS	↑↑ miR-590-3p: ↓ proliferation via partially decreasing CDK4	[315]
	miR-338-3p, RUNX2, CDK4 and MAPK pathway	MG-63, U2OS and hFOB	↑↑ miR-338-3p: ↓ cell viability and colony formation, migration, and invasion, but ↑ apoptosis via targeting RUNX2 and CDK4 and inhibiting the MAPK pathway	[316]
	91 H, CDK4, Cyclin D1, and PCNA	MG63 and U2OS	∆ 91 H: ↓ proliferation, migration and invasion, but ↑ apoptosis via inducing methylation of CDK4 promoter and downregulating Cyclin D1, PCNA and CDK4	[317]
Ovarian cancer	CDK4/6	CD8 + T cells and B cells	CDK4/6 inhibition and anti-PD-1 antibody: ↑ efficacy of anti-PD-1 therapy and immune infiltration	[318]
	LRRC75A-AS1-hsa-miR-330-5p/CDK4/6 axis, IFN-γ, ISG response, and STING pathway	OVCAR3 and HOC7	Palbociclib: ↑ secretion of IFN-γ and ↑ ISG response, ↑ expression of antigen-presenting molecules; via STING pathway LRRC75A-AS1-hsa-miR-330-5p/CDK4/6 axis is involved in inhibiting the immune response of OC patients	[319]
	CDK4/6-p-Rb signaling pathway, COL6A3	OCSPCs, epi-OCSPCs, msc-OCSPCs, SKOV3, ES2TR and ES2	∆ COL6A3: ↓ expression of DNMT1, CDK4, CDK6, and p-Rb and ↓ formation, invasion, tumor growth, and metastasis	[320]
	CDK4/6 and PARP	OVCAR5 and SKOV3	CDK4/6 and PARP dual inhibitor, ZC-22: ↑ cell cycle arrest and ↑ DNA damage The efficacy of ZC-22 was found to be higher than the combination of PARPi Olaparib and CDK4/6i Abemaciclib	[217]
	miR-506-CDK4/6-FOXM1 axis	SKOV3, HeyA8	↑↑ miR-506: ↓ proliferation via targeting CDK4/6-FOXM1 axis	[321]
Pancreatic Adenocarcinoma	CDK4/6	Mia-Paca-2, Hs766t and PL-45	∆ CDK4/6: ↑ defective DNA repair by homologous recombination after chromosomal damage	[322]
	CDK4/6-E2 F1 signaling pathway, MAGED1, FBP1	PANC-1 and BxPC-	PD0332991, CDK4/6 inhibitor, was found to stabilize FBP1 to hinder aerobic glycolysis MAGED1, the key mediator in the CDK4-induced destabilization of FBP1, was repressed by PD0332991	[323]
	CDK4/6, MEK, ERK and Rb	BxPC-3, MiaPaCa-2, Panc-1, CFPAC, Panc 10.05, HPNE-*KRAS*, and HPNE	Combination of MEK and CDK4/6 inhibition: ↓ ERK and Rb phosphorylation and ↓ proliferation	[324]
Pancreatic cancer	miR-143, miR-506, CDK1, CDK4, and CDK6	HFL-1, MIA-Paca-2, and Panc-1	Combinatorial treatment with miR-143 and miR-506: ↓ CDK1, CDK4, and CDK6, cell growth	[51]
	miR-196a/ NFKBIA axis and Cyclin D1 and CDK4/6	PANC-1, Capan-2, BxPC-3, SW1990, and H6C7	∆ miR-196a: ↓ proliferation, due to G0/G1 arrest via downregulating Cyclin D1 and CDK4/6 expression and ↓ migration NFKBIA was a direct target of miR-196a The expressions of Cyclin D1 and CDK4/6 were increased after silencing NFKBIA	[325]
Papillary thyroid cancer	miR-1256/HTR3A axis and CDK4 and Cyclin D, and p21	TPC-1, B-CPAP and GLAG-66 and Nthy-ori-3–1	↑↑ miR-1256: ↓ proliferation and ↑ cell cycle G0/G1 phase arrest via targeting HTR3A and regulating CDK4 and Cyclin D, and p21	[326]
Prostate cancer	miR-3619-5p/CDKN1A axis and cyclin D1, CDK4/CDK6 and p21	DU145, PC3, LNcaP and RWPE-1	↑↑ miR-3619-5p: ↓ cell growth via activating p21 expression miR-3619-5p induces CDKN1A expression via directly interacting the promoter, thus regulates prostate cancer cell cycle-associated genes including cyclin D1, CDK4/CDK6	[327]
	miR-96/ FOXF2 axis and CyclinA1, CDK2 and CDK4	LNCaP, PC-3 and DU-145	∆ miR-96: ↓ proliferation and cell cycle progression via upregulating FOXF2 and downregulating CyclinA1, CDK2 and CDK4 FOXF2 was a direct target of miR-96	[328]
	NR2F2-AS1 and CDK4	22Rv1	↑↑ NR2F2-AS1: ↑ proliferation and cell cycle progression via upregulating CDK4	[329]
Skin cancer	CDK4/6, Rb, cyclin D	A431 and A375	CDK4/6 inhibitor, Rafoxanide: ↓ viability, expression of CDK4/6, Rb, cyclin D, pho-CDK4/6 and pho-Rb, and ↑ G1 phase arrest and apoptosis	[330]
Uveal melanoma	RB, HGF, CDK4/6	UM001, UM002B, and UM004	Abemaciclib, CDK4/6 inhibitor: ↑ G1 arrest and ↓ cell growth in Merestinib and Abemaciclib combination: ↓ HGF-mediated protection from cellular senescence HGF decreased the growth-inhibitory effect of Abemaciclib	[331]
	CDK4/6, MEK-ERK signaling pathway, OxPhos pathway	UM001, UM004, OMM1.3, WM3618F, and 92.1 cells	Combination of MEK plus CDK4/6 inhibition: ↓ cell cycle arrest but does not induce apoptosis Upregulation of OxPhos pathway was observed in both MEKi-resistant tumors and CDK4/6i-tolerant tumors	[332]

∆ knock-down, deletion or inhibition, *PFS* progression-free survival, *HR* homologous recombination, *TMZ* temozolomide, *CDDP* Cisplatin, *CDK4/6i* inhibitors targeting CDK4/6, *PDAC* Pancreatic ductal adenocarcinoma, *OC* Ovarian cancer, *LAR* Luminal Androgen Receptor, *TNBC* triple negative breast cancer

### Animal studies

Experiments in animal models of AML have verified that CDK4/6 inhibition enhances autophagy. Moreover, concurrent administration CDK4/6 inhibitor and autophagy inhibitor has reduced tumor growth in these models [333]. Similarly, combination of cisplatin and CDK4/6 inhibitors has significantly reduced bladder cancer growth [195]. In xenograft models of breast cancer, CDK4/6 inhibitors could reduce proliferation, and enhance anti-tumor immune responses [198]. In addition, in this type of cancer, combined inhibition of CDK2 and CDK4/6 has enhaced sensitivity to palbociclib [98]. Besides, combination of CDK4/6 inhibitor, abemaciclib, with c-Met/Trk inhibitor, altiratinib has been shown to be effective against glioma-initiating cells [256]. Table 11 shows function of CDK4/6 in animal models of cancer.

**Table 11:** Function of CDK4/6 in animal models of cancer

Tumor Type	Animal models	Results	References
Acute myeloid leukemia	NOD/Shi-scid IL2Rgnull (NOG) mice	CDK4/6 inhibition: ↑ autophagy Combination of CDK4/6 inhibition and autophagy inhibitor, chloroquine: ↓ tumor growth	[333]
	4–6-week-old BALB/c nude mice	↑↑ miR-362-5p: ↑ tumor growth	[202]
Bladder cancer	mice	CDK4/6 inhibition and CDDP combination: ↓ tumor growth	[195]
	6-week-old BALB/c-A nude mice	↑↑ miR-124: ↓ tumor growth	[203]
Breast cancer	6–7-week-old female FVB MMTV-PyMT, Balb/c (), and 8-week-old Foxn1nu mice	∆ CDK4/6: ↓ proliferation, ↑ anti-tumor immunity and cell cycle arrest	[198]
	female nude mice	∆ PTEN: ↑ clinical cross-resistance to CDK4/6 and PI3Kα inhibitors via increased AKT activation	[172]
	7-week-old female NOG CIEA mice	∆ CDK4/6 and AKT: ↓ tumor growth of ER + breast xenografts resistant to fulvestrant	[207]
	6- to 8-week-old female NSG mice 6- to 8-week-old female immune-competent C57BL/6 mice	Combined PI3Kα and CDK4/6 inhibition: ↑ activation of tumor-infiltrating T-cell and cytotoxicity and ↓ immunosuppressive myeloid-derived suppressor cells	[208]
	4-week-old BALB/c nude mice	Combined Inhibition of CDK2 and CDK4/6: ↓ resistance to Palbociclib	[98]
	6-week-old female NOD-SCID mice	PARPi olaparib and the CDK4/6i palbociclib: ↓ tumor growth	[209]
	6-week-old female BALB/c nude mice	∆ CDK4/6: ↓ tumor metastasis by destabilizing the ZEB1 protein ∆ USP51: ↓ tumor metastasis through the regulation of ZEB1	[210]
	6-week-old CD-1 athymic nude mice	Blocking AKT/S6 signaling by targeting PI3K was found to be effective in blocking proliferation of palbociclib-resistant cells	[214]
	6-week-old female athymic nude mice	CDK4/6 and PARP dual inhibitor, ZC-22: ↑ cell cycle arrest and ↑ DNA damage more than the combination of Olaparib and Abemaciclib, and ↑ response to Cisplatin	[217]
	Female BALB nude mice	abemaciclib and ABT-263 combination: ↓ tumor growgh	[218]
	4-week-old BALB/c nude mice	↑↑ miR-124: ↓ tumor growth	[219]
	4-week-old nude mice	∆ MALAT1: ↑ inhibitory effect of miR-124 on the tumor growth	[222]
Cervical cancer	4–5-week-old male BALB/c nude mice	∆ circ_0000326: ↓ tumor growth	[227]
Clear cell renal cell carcinoma	4–5-week-old male BALB/c nude mice	↑↑ miR-1: ↓ tumor growth	[228]
	NOD/SCID/IL2Rγ-null (NSG) mice	∆ DMDRMR: ↓ tumor growth	[229]
	4–5-week-old male BALB/c nude mice	↑↑ miR-206: ↓ tumor size and weigh	[230]
Colon cancer	Male athymic BALB/c nude mice	∆ HAGLR: ↓ tumor growth	[231]
	6-week-old female Balb/c nude mice	↑↑ miRNA-20b-5p: ↓ tumor growth	[232]
Colorectal cancer	6-week-old BALB/c athymic nude mice	↑↑ MCM3AP-AS1: ↑ tumor growth	[233]
	5–6-week-old male BALB/c nude mice	↑↑ miR-142-3p: ↓ tumor growth	[234]
	4–6-week-old male BALB/c athymic nude mice	↑↑ miR-875-5p: ↓ tumor growth	[236]
Esophageal squamous cell carcinoma	4–5-week-old female BALB/c athymic nude mice	↑↑ miR-1: ↓ tumor growth	[243]
Ewing sarcoma	7–8 week old nude female mice	Combination of CDK4/6 and IGF1R inhibition: ↑ survival and ↓ tumor progression	[173]
Gastric cancer	4-week-old BALB/c nude mice	↑↑ miR-1301-3p: ↑ tumor growth	[247]
	6-week-old female BALB/c nude mice	∆ Linc-ROR: ↓ tumor growth	[252]
	4–week-old female BALB/c nude mice	∆ GCRL1: ↓ tumor growth, tumor size, and weight	[254]
Glioblastoma	6–8 week old SCID Ncr mice	Palbociclib, CDK4/6 inhibitor: ↑ survival	[255]
	6-to-8-week-old female BALB/c SCID NCr mice	Combination of CDK4/6 inhibitor, abemaciclib, with c-Met/Trk inhibitor, altiratinib was effective against GICs	[256]
Glioblastoma multiforme	BALB/C nu/nu nude mice	CDA-2 treatment: ↑ radiosensitivity which acts like the effect of miR-124 restoration and CDK4 knockdown	[258]
	4–5-week-old female BALB/c nude mice	↑↑ miR-138: ↓ tumor growth	[259]
	4-week-old male nude mice	∆ circMMP9: ↓ tumor growth	[260]
Glioma	5-week-old female BALB/c nude mice	Combination of TMZ and abemaciclib treatment showed antitumor efficacy	[261]
	4-week-old male BALB/c nude mice	Sevoflurane treatment: ↓ tumor volume and weight via reducing HMMR-AS1	[262]
H. pylori related gastric cancer	4–6-week-old male BALB/c nude mice	↑↑ miR-101: ↓ tumor growth	[263]
Head and neck squamous cell carcinoma	nude mice	Combination of CDK4/6 inhibitor, LY2835219, and metformin: ↓ tumor growth	[265]
Hepatocellular carcinoma	4–5-week-old female BALB/C nude mice	Aminoquinol, a new CDK4/6 and PI3K/AKT inhibitor: ↓ tumor growth	[174]
	6–8-week-old BALB/c, all-female nude mice	∆ circ_0001588: ↓ tumor size, volume and weight	[267]
	4-week-old male BALB/c nude mice	∆ hsa_circ_0016788: ↓ tumor growth	[268]
	6-week male Bl6/Rag2/GammaC double knockout nude mice	∆ CCDC144NL-AS1/WDR5 or ↑↑ miR-940: ↓ tumor growth	[270]
	4-week-old female BALB/c nude mice	∆ circSP3: ↓ tumor volume and weight	[273]
	BALB/c nude mice	∆ VPS9D1-AS1: ↓ tumor growth	[24]
Kaposi’s sarcoma–associated herpesvirus	4–6-week-old female BALB/c nude mice	↑↑ miR-34a-5p: ↓ tumor volume and weight	[274]
Lung cancer	female athymic BALB/c nude mice	∆ LINC01194: ↓ tumor volume and weight	[278]
	6-week-old male BALB/c nude mice	↑↑ hsa_circ_0014235: ↑ DDP chemoresistance	[279]
	5–6-week-old male BALB/c nude mice	↑↑ miR-613: ↓ tumor growth	[280]
	4-week-old female BALB/c nude mice	↑↑ miR-340: ↓ tumor growth	[283]
	4–6-week-old male BALB/c athymic nude mice	↑↑ miR-326: ↑ tumor volume and weight	[285]
	4–6-week-old male BALB/c athymic nude mice	↑↑ miR-134: ↓ tumor growth	[285]
	male athymic BALB/c nude mice	∆ lncSENCR: ↓ tumor growth	[291]
	5–6-week-old BALB/c athymic nude mice	↑↑ miR-545: ↓ tumor volume and weight	[292]
Medulloblastoma	Balb/C nude mice	∆ HOTAIR: ↓ tumor growth	[296]
Melanoma	6–7-week-old female BALB/c nude mice	Palbociclib and GSK3326595 treatment: ↓ tumor volume ∆ PRMT5: ↓ emergence of CDK4/6 inhibitor resistance In Vivo	[298]
	CrTac:NCr-Foxn1nu mice	Combination of MEK and CDK4/6 inhibitors: ↓ tumor size in NRAS mutant cells	[300]
	7–8 weeks old female, pathogen free C.B 17-Scid mice	∆ CDK4 or CDK6: ↓ tumor growth CDK4/6 inhibitor, PD0332991: ↓ tumor growth	[301]
	Male C57BL/6 mice (Jackson Labs) and NSG mice	Combination of MEK and CDK4/6 inhibitors was more effective at postponing regrowth of mutant BRAF melanoma in immunocompetent versus immune-deficient mice	[302]
	nude mice	∆ hsa_circ_0025039: ↓ tumor volume and weight	[304]
Nasopharyngeal carcinoma	4–week-old BALB/c nude male mice	∆ RP11-624L4.1: ↓ tumor growth	[61]
Oral squamous cell carcinoma	4–6-week-old male BALB/c nude mice	↑↑ miR-198: ↓ tumor size and volume	[312]
Osteosarcoma	6–8-week-old BALB/c nude mice	∆ 91 H: ↓ tumor growth	[317]
Ovarian cancer	6-week-old female C57BL/6 mice	Abemaciclib (inhibitor of CDK4/6) treatment: ↓ tumor growth and ↑ proinflammatory immune response	[318]
	6–8-week-old female C57BL/6J mice	CDK4/6 Inhibitor, palbociclib: ↓ tumor growth by activating the immune microenvironment	[319]
	Female BALB/cAnN.Cg-*Foxn1nu*/CrlNarl null mice	∆ COL6A3: ↓ metastasis and tumor growth via regulating CDK4/6 and p-Rb	[320]
	6-week-old female athymic nude mice	CDK4/6 and PARP dual inhibitor, ZC-22: ↑ response to Cisplatin	[217]
	mice	↑↑ miR-506: ↓ proliferation	[321]
Pancreatic Adenocarcinoma	6–8-week-old female athymic nude mice	∆ CDK4/6: ↓ tumor growth	[322]
Pancreatic ductal adenocarcinoma	4–5-week-old athymic nude mice	Combination of MEK and CDK4/6 inhibition: ↓ tumor growth and ↑ overall survival	[324]
Skin cancer	female BALB/C nude mice	CDK4/6 inhibitor, Rafoxanide: ↓ tumor growth	[330]
Uveal melanoma	NSG-hHGFki mice	Merestinib and Abemaciclib combination: ↓ tumor growth in NSG-hHGFki mice	[331]
	6–8 week-old athymic (nu/nu) homozygous nude mice	CDK4/6 inhibition: ↑ cytostasis and ↓ tumor growth as effective as MEKi plus CDK4/6i treatment	[332]

∆ knock-down or deletion, *NSG* Nod SCID γ, *NSG-hHGFki* NOD.Cg-Hgftm1.1(HGF)Aveo Prkdcscid IL2rgtm1Wjl/J, *GICs* glioma-initiating cells

### Investigations in clinical samples

Investigations in breast cancer samples have shown up-regulation of CDK4/6 in different subtypes. For instance, CDK6 levels have been found to be higher in FAT1-deleted samples compared with those having wildtype FAT1 [196]. Another study has shown up-regulation of CDK4/6 and pRb levels in HER2 + breast cancer samples [334]. In ovarian cancer samples, up-regulation of CDK6 has been associated with shorted OS and immunosuppressive state [319]. Moreover, in this type of cancer, up-regulation of a functional counterpart of CDK4/6, i.e. COL6A3 has been associated with shorter OS and advanced clinical stage [330]. Table 12 shows dysregulation of CDK4/6 in clinical samples.

**Table 12:** Dysregulation of CDK4/6 in clinical samples

Tumor type	Samples	Expression (Tumor vs. Normal)	Kaplan–Meier analysis (impact of regulators dysregulation)	Multivariate Cox regression analysis	Association of dysregulation of regulators with clinicopathologic characteristics	References
Acute myeloid leukemia	24 patients with AML and normal controls	Up of miR-362-5p (which indirectly regulated CDK4)	_	_	_	[202]
Bladder cancer	27 tumor tissues	Up of CDK4 and down of miR-124	_	_	_	[203]
	25 PTANC	Down of miR-195 (which suppressed CDK4)	Poor OS	_	_	[204]
	83 PTANC	Down of miR-124 (which suppressed CDK4)	Poor OS	_	_	[205]
Breast cancer	TCGA dataset	Up-regulation of CDK6 in FAT1-deleted samples than those in FAT1 wild-type samples up-regulation of CDK4 than CDK6 transcripts in most ER + breast cancers but not in FAT1 negative tumors	_	_	_	[196]
	77 cases of HER2 + and 53 cases of HER2- breast cancer	Up-regulation of CDK4/6 and pRb levels in HER2 +	_	_	_	[334]
	GEO database (GSE4922, GSE6532, GSE20194, GSE26459, GSE98987)	Up-regulation of HMGB1 in tamoxifen‐resistant group	Shorter PFS for HR + BC patients with endocrine therapy after surgery			[215]
	40 PTANC	Down of miR-124 (which suppressed CDK4)	_	_	_	[219]
	40 PTANC	Down of miR-124 (which suppressed CDK4)	Poor OS	Expression of miR-124 was found to be correlated with poor survival	advanced pathological stages	[222]
	60 PTANC	Down of miR-1301-3p (which indirectly suppressed CDK4)	_	_	tumor size and clinical stage	[224]
	TCGA dataset: 658 tumor and 86 normal breast tissue	Down of miR-34c (which suppressed CDK4)	_	_	_	[226]
Cervical cancer	GEO database (GSE102686) 60 PTANC	Up of circ_0000326 (which indirectly regulated CDK4)	_	_	_	[227]
Clear cell renal cell carcinoma	41 PTANC 90 PTANC	Down of miR-1 (which suppressed CDK4)	Poor OS	_	clinical Stage and T classification	[228]
	TCGA dataset	Up of DMDRMR (which indirectly regulated CDK4)	Poor OS	_	pathologic stage, tumor size, metastatic status, and Fuhrman grade	[229]
	41 PTANC	Down of miR-206 (which directly suppressed CDK4)	_	_	_	[230]
Colon cancer	25 PTANC	Up of HAGLR circMMP9 (which regulated CDK4 and CDK6)	_	_	_	[231]
Colorectal cancer	60 PTANC	Up of MCM3AP-AS1 (which regulated CDK4)	Poor OS	_	_	[233]
	116 PTANC	Down of miR-142-3p (which suppressed CDK4)	Poor OS	_	_	[234]
	TCGA dataset	Up of CDK4/6	_	_	_	[235]
	3 patients with mild myelosuppression and 3 with severe myelosuppression and (MildA, MildB, SevereA, and SevereB groups)	Up of miR-122-5p (which suppressed CDK4) in the SevereB and MildB groups than SevereA and MildA groups	_	_	severity of myelosuppression caused by chemotherapy	[335]
	92 PTANC	Down of miR-875-5p (which indirectly suppressed CDK4)	Poor OS	_	tumor size, differentiation, TNM stage, and lymph node metastasis	[236]
	GSE167326: 150 PTANC	Down of uc.77- (which indirectly suppressed CDK4)	_	_	_	[237]
	67 PTANC	Up of LINC00665 (which indirectly regulated CDK4)	_	_	_	[238]
	10 PTANC	Down of miR-29a-3p (which indirectly regulated CDK4)	_	_	_	[239]
Epithelial ovarian cancer	32 patients and 20 controls	Up of PCAT-1 (which upregulated CDK4)	_	_	larger tumor sizes and advanced tumor grades	[240]
Esophageal cancer	20 PTANC	Up of CDK4 and Down of miR-486	_	_	_	[241]
	18 PTANC	Down of miR-124 (which suppressed CDK4)	_	_	_	[242]
Esophageal squamous cell carcinoma	34 PTANC	Up of CDK4 and Down of miR-1 (which suppressed CDK4)	_	_	_	[243]
Ewing's sarcoma	Ewing’s sarcoma patients and normal controls	Up of DLX6-AS1 (which regulated CDK4)	_	_	_	[244]
Gastric cancer	TCGA dataset: 446 tumor tissues and 15 normal tissues 60 PTANC	Up of miR-1301-3p(which indirectly upregulated CDK4)	_	_	_	[247]
	27 PTANC	Up of Linc-ROR (which indirectly upregulated CDK4)	Poor OS	_	_	[252]
	50 PTANC	Down of miR-29a-3p (which indirectly suppressed CDK4)	_	_	_	[253]
	GEO dataset 26 tumor tissues and 14 normal tissues	Up of GCRL1 (which regulated CDK4)	_	_	_	[254]
Glioblastoma multiforme	87 glioblastoma multiforme tissue samples	Up of CDK4	_	_	radio-resistance	[258]
	25 tumor tissues and 14 normal tissues TCGA dataset	Down of miR-138(which indirectly suppressed CDK4)	Poor OS and PFS	_	_	[259]
	18 PTANC	Up of circMMP9 (which regulated CDK4)	_	_	_	[260]
Glioma	12 glioma tissues of high grade and 6 normal tissues	Up-regulation of CDK4	_	_	_	[261]
	37 tumor tissues and 10 normal tissues	Up of HMMR-AS1 (which indirectly regulated CDK4)	Poor OS	_	advanced stage	[262]
H. pylori related gastric cancer	50 pairs of H. pylori positive and negative tissues	Down of miR-101 in H. pylori infected tissues (which indirectly suppressed CDK4)	_	_	_	[263]
Head and neck mucosal melanoma	29 HNMM tissue samples (16 OMM and 13 SNMM)	Up-regulation of CDK4 in five samples (up-regulation in OMM samples than in SNMM) samples	_	_	_	[264]
Hepatocellular carcinoma	63 PTANC and 40 healthy controls	Up of hsa_circ_0016788 (which regulated CDK4)	_	_	_	[268]
	135 PTANC	Up of CCDC144NL-AS1 (which indirectly regulated CDK4)	Poor OS	_	HBV and HCV infection, cirrhosis state, differentiation state, T stage, and the N stage of patients	[270]
	48 PTANC	Up of circSP3 (which regulated CDK4)	_	_	tumor size and TNM stage	[273]
	GEO database (GSE65485) and TCGA dataset 80 PTANC	Up of VPS9D1-AS1 (which indirectly upregulated CDK4)	Poor OS	_	tumor size and more advanced tumor, TNM stage	[24]
Leiomyosarcoma	a larger cohort of 99 patients with 159 tumor samples	Up-regulation of CDK4/6 (92 were positive for CDK4, 138 for CDK6)	_	_	_	[275]
Lung cancer	26 PTANC	Up of LINC01194 (which regulated CDK4)	_	_	gender, tumor size, TNM stage and lymph node metastasis	[278]
	35 PTANC	Up of hsa_circ_0014235 (which regulated CDK4 and CDK6)	_	_	_	[279]
	56 PTANC 38 PTANC	Down of miR-613 (which suppressed CDK4)	Poor OS	_	_	[280]
	GEO database (GSE64591: 100 PTANC)	Down of miR-34b-3p (which suppressed CDK4)	_	_	_	[281]
	11 PTANC	Up of CDK4 and circRNA_001010	_	_	_	[282]
	64 PTANC	Down of miR-340 (which suppressed CDK4)	Poor OS	_	lymph node metastasis, larger tumor size, advanced TNM stage and poor prognosis	[283]
	38 PTANC	Up of CDK4	_	_	tumor stage	[284]
	39 PTANC	Down of miR-326 (which indirectly suppressed CDK4)	Poor OS	_	_	[285]
	39 PTANC	Down of miR-134 (which indirectly suppressed CDK4)	Poor OS	_	tumor size, smoking history, TNM stage, and lymph node metastasis	[285]
	71 PTANC	Down of miR-98 (which indirectly suppressed CDK4)	Poor OS	_	_	[286]
	41 PTANC	up of miR-1290 (which indirectly upregulated CDK4)	_	_	lymph node metastasis and advanced tumor stage	[287]
	15 PTANC	Up of CDK4 and Up of circHIPK3 (which indirectly upregulated CDK4)	_	_	_	[288]
	80 PTANC	Down of miR-593 (which indirectly suppressed CDK4)	Poor OS	_	tumor size, lymph node metastasis, distant metastasis, and advanced pathological TNM stage	[289]
	30 PTANC	Up of lncSENCR (which indirectly regulated CDK4)	_	_	_	[291]
	15 PTANC 10 PTANC	Down of miR-545 (which directly suppressed CDK4)	_	_	_	[292]
	32 PTANC	Down of linc00703 (which affected expression of CDK4)	_	_	_	[293]
Melanoma	3 PTANC 43 tumor tissues 18 PTANC	Up of hsa_circ_0025039 (which regulated CDK4)	Poor OS	_	pathological node status, pathological metastasis status and clinical stage	[304]
	36 melanoma patients and 16 healthy controls	Down of miR-206 (which directly suppressed CDK4)	_	_	_	[305]
Multiple myeloma	28 tumor tissues and 15 healthy controls	Down of miR-338-3p (which directly suppressed CDK4)	_	_	_	[307]
Nasopharyngeal carcinoma	56 PTANC	Up of CDK4 and Down of miR-539 (which suppressed CDK4)	_	_	_	[310]
	7 NPC and 7 normal NPE tissues 20 NPC samples and 14 inflammatory NPE samples 130 tumor samples	Up of RP11-624L4.1 (which interacted with CDK4)	Poor OS and DFS	RP11-624L4.1 expression, clinical stage, N stage, M stage, T stage were correlated with OS	T stage, N stage, M stage, clinical stage, survival state, and relapse	[61]
Oral squamous cell carcinoma	24 PTANC	Up of MMP1 (which regulated CDK4)	_	_	_	[311]
	80 PTANC	Up of CDK4 and Down of miR-198 (which suppressed CDK4)	Poor OS and DFS	_	_	[312]
	45 PTANC	Down of miR-519d-3p (which indirectly suppressed CDK4)	_	_	higher tumor grade	[313]
	10 PTANC	Down of miR-9	_	_	_	[314]
Osteosarcoma	5 PTANC	Up of 91 H (which affected the methylation of CDK4 promoter)	_	_	_	[317]
Ovarian cancer	TCGA, GEO and GTEx databases	Up-regulation of CDK4/6	Shorter OS for higher expression of CDK6	_	immunosuppressive state of OC	[319]
	TCGA dataset (n = 369)	Up-regulation of COL6A3 (which regulated CDK4/6)	Shorter OS	_	advanced-stage carcinoma	[330]
	92 patients	Up of CDK4 and Down of miR-506 (which suppressed CDK4)	_	_	_	[321]
Papillary thyroid cancer	49 PTANC	Down of miR-1256 (which indirectly regulated CDK4)	_	_	tumor size and TNM stage	[326]
Prostate cancer	73 PTANC	Up of miR-96 (which indirectly upregulated CDK4)	_	_	higher PSA level, lymph node metastasis, pathologic stage and distant metastasis	[328]
	60 PTANC	Up of CDK4 and Up of NR2F2-AS1 (which upregulated CDK4)	Poor OS	_	_	[329]

(PTANC: pairs of tumor samples and adjacent non-cancerous samples, PFS: progression-free survival, OS: overall survival, TCGA: Cancer Genome Atlas, GEO: Gene Expression Omnibus, GTEx: Genotype Tissue Expression, OC: Ovarian cancer, OMM: oral mucosal melanoma, SNMM: nasal cavity/sinuses melanoma)

A number of clinical studies have evaluated the effects of CDK4/6 inhibition on survival of patients (Table 13). For instance, treatment of 22 breast cancer patients with a CDK4/6 inhibitor has resulted in complete response in one patient, partial response in 8 patients, and stable disease in 13 patients [336]. Another study in breast cancer patients has indicated better progression-free survival time in those treated with CDK4/6 inhibitors than those received PI3K inhibitors. Moreover, Combination of CDK4/6 inhibitors and endocrine therapy has yielded better OS than PI3K/mTOR inhibitors [337]. Promising results have also obtained from studies in other types of cancers.

**Table 13:** Effects of CDK4/6 inhibitors or other therapeutic agents in clinical settings

Tumor type	Samples	Inhibitors / Therapy	Function	References
Breast cancer	22 patients	CDK4/6 inhibitors	After 18 months CDK4/6 treatment, best objective response was complete response in 1, partial response in 8, and stable disease in 13 patients	[336]
	9771 patients	CDK4/6 inhibitors, PI3K inhibitor, and endocrine therapy	PFS was better in CDK4/6 inhibitors than PI3K inhibitors Combination of CDK4/6 inhibitors and endocrine therapy could increase OS than PI3K/mTOR inhibitors	[337]
	3421 breast cancer patients	endocrine therapy and CDK4/6 inhibitors	In comparison with endocrine therapy alone, adding CDK4/6 inhibitors enhanced OS in patients with HR-positive, HER2-negative metastatic breast cancer But, adding of CDK4/6 inhibitors also increased the incidences of grade 3–4 adverse events	[338]
Breast cancer	2968 patients	CDK4/6 inhibitors	Treatment with CDK4/6 inhibitors was found to be worse in patients with gBRCAm mBC than those with gBRCAwt and unknown gBRCA status	[339]
	71 patients	CDK4/6 inhibitors	A higher median value of Ki67 was observed in cases with second-line treatment, while the luminal B subtype was more prevalent. Luminal A subtype was correlated with a longer PFS. A higher continuous Ki67 value was correlated with shorter PFS. Luminal B subtype had a significantly worse outcome. PFS in patients with endocrine therapy in combination with CDK4/6i was inversely correlated with Ki67 expression but not with PR	[340]
	43 patients, (17 prior CDK4/6i exposure)	CDK4/6 inhibitors, combination of EVE and EXE	No significant difference was found in PFS or OS between patients who had not received prior CDK4/6is and those who had	[341]
	3182 patients	CDK4/6 inhibitors	CDK4/6 inhibitors could increase PFS in patients with HR-positive/ HER2-negative advanced breast cancer	[342]
	ongoing phase II trial (NCT02308020) (pre-treated patients with CNS metastases) (including total 52 patients with HR + /HER2- CNS metastases are currently available)	CDK4/6 inhibitor (abemaciclib)	There was scarcity of data pertaining to the development of new CNS metastases	[343]
Breast cancer	130 HR + BC patients and 83 endocrine‐resistant breast cancer patients	CDK4/6 inhibitors plus endocrine therapy	Patients receiving CDK4/6 inhibitors and endocrine therapy in the HMGB1‐positive group showed improved PFS in comparison with those in the HMGB1‐negative group	[215]
	30 patients	CDK4/6 inhibitors plus hormonal therapy	Patients had a PIK3CA mutation at the baseline of CDK4/6i treatment had a shorter PFS, in comparison with patients without mutation PIK3CA mutations were found to be predict response to CDK4/6i	[344]
	2799 patients	CDK4/6 inhibitors (palbociclib, ribociclib, abemaciclib)	Three inhibitors showed comparable efficacy, but they had differences in safety and tolerability. Abemaciclib showed worse tolerability with higher treatment discontinuation because of GI toxicity	[345]
	160 patients (185 treatment occurrences)	PI3K/mTOR/CDK4/6 inhibitors	Inhibition of PI3K/mTOR/CDK4/6 could have an effect on the development of edema, so could cause or exacerbate progression of BCRL in patients with MBC	[346]

*gBRCAm* mutated gBRCA, *mBC* metastatic breast cancer, *gBRCAwt* wild type gBRCA, *EVE* everolimus and *EXE* exemestane, *PFS* progression‐free survival, *BCRL* breast cancer-related lymphedema, *MBC* metastatic breast cancer

## Discussion

Expression and activity of CDKs have been assessed in animal models of cancers, cell lines and clinical samples of patients having different types of cancers. CDK1 and CDK2 are the most comprehensively assessed members of this family. Additionally, a number of studies have addressed involvement of CDKs 3, 4/6, 5, 7 and 9 in cancer cell lines. Other members of this protein family have not been thoroughly assessed.

The above-mentioned studies have revealed a number of CDKs-interacting molecules including mRNA coding genes as well as lncRNAs and miRNAs. PVT1, NCK1-AS1, FOXD2-AS1, SNHG4, SNORD52, TMPO-AS1, TONSL-AS1, DLEU1 and CASC11 are among lncRNAs that interact with CDKs. Meanwhile, miR-378a-5p, miR-34c-3p, miR-181a, miR-195-3p and miR-205 have been shown to regulate expression of certain CDKs through binding with the 3'UTR of their transcripts. Since miRNAs can efficiently reduce expression of CDKs, identification of additional CDKs-targeting miRNAs through in silico and experimental methods can facilitate design of novel treatment modalities for cancers. Moreover, available data indicate that expressions of CDKs are regulated through a complex regulatory network consisted of both genetic and epigenetic mechanisms which can be dysregulated during the course of cancer evolution. Application of various quantitative experimental and computational methods in a "system biology" approach is needed to unravel complicated aspects of the mentioned network and develop novel modalities to combat cancer-a prototype of disorders associated with dysregulation of CDKs.

## Conclusion

Since activity of CDKs is associated with induction of stem cell properties, drugs targeting these proteins might be used for effective elimination of cancer stem cells and reduction of tumor metastases. This implicates that CDKs are involved in the pathogenesis of a high spectrum of cancers, including different types of carcinomas as well as non-epithelial malignancies. Coming from this point of view CDKs will come more and more in the focus as therapeutical targets.

Activity levels of CDKs can be used for prediction of cancer prognosis and response of patients to various therapeutic options. In fact, an appropriate approach for implementation of personalized medicine in the field of cancer therapy is measurement of activity of these proteins.

Cumulatively, CDKs represent ideal therapeutic targets for cancer. Thus, future studies should focus on assessment of their activities in different tumors and identification of their association with clinicopathological data. Moreover, the presence of putative genetic variants within *CDK* coding genes might affect their activity and susceptibility of persons to different cancers. This note should also be assessed in future studies.

## Data Availability

The analyzed data sets generated during the study are available from the corresponding author on reasonable request.
